# *Metarhizium*: jack of all trades, master of many

**DOI:** 10.1098/rsob.200307

**Published:** 2020-12-09

**Authors:** Raymond J. St. Leger, Jonathan B. Wang

**Affiliations:** Department of Entomology, University of Maryland, College Park, MD, USA

**Keywords:** *Metarhizium* and *Pochonia chlamydosporia*, plant endophyte and symbiont, insect killing (entomopathogen), virulence evolution (host switching and speciation), sexual, parasexual and asexual life histories (Red Queen and Vicar of Bray), parasitism to insects or nematodes

## Abstract

The genus *Metarhizium* and *Pochonia chlamydosporia* comprise a monophyletic clade of highly abundant globally distributed fungi that can transition between long-term beneficial associations with plants to transitory pathogenic associations with frequently encountered protozoans, nematodes or insects. Some very common ‘specialist generalist’ species are adapted to particular soil and plant ecologies, but can overpower a wide spectrum of insects with numerous enzymes and toxins that result from extensive gene duplications made possible by loss of meiosis and associated genome defence mechanisms. These species use parasexuality instead of sex to combine beneficial mutations from separate clonal individuals into one genome (Vicar of Bray dynamics). More weakly endophytic species which kill a narrow range of insects retain sexuality to facilitate host–pathogen coevolution (Red Queen dynamics). *Metarhizium* species can fit into numerous environments because they are very flexible at the genetic, physiological and ecological levels, providing tractable models to address how new mechanisms for econutritional heterogeneity, host switching and virulence are acquired and relate to diverse sexual life histories and speciation. Many new molecules and functions have been discovered that underpin *Metarhizium* associations, and have furthered our understanding of the crucial ecology of these fungi in multiple habitats.

## 1. Introduction

‘The vivacious vicar living under King Henry VIII, King Edward VI, Queen Mary and Queen Elizabeth, was first a Papist, then a Protestant, then a Papist, then a Protestant again. He had seen some martyrs burnt (two miles off) at Windsor and found this fire too hot for his tender temper. This vicar, being taxed [attacked] by one for being a turncoat and an inconstant changeling, said, ‘Not so, for I always kept my principle, which is this—to live and die the Vicar of Bray.’—*Worthies of England*, Thomas Fuller, published 1662.

*Metarhizium* is a genus of highly abundant fungi with several identities. They are best known for their ability to infect and kill many different arthropods, but most are also saprophytes, rhizosphere colonizers and beneficial root endophytes, with the ability to switch between these different lifestyles. Furthermore, *Metarhizium* forms a monophyletic clade with the nematode pathogen *Pochonia chlamydosporia* and *Metarhizium* representatives have transitioned to fungal and even lizard hosts. Aside from playing a crucial role in natural ecosystems (the fungal kingdom is responsible for a large proportion of insect disease), entomopathogenic *Metarhizium* species and nematophagous *P. chlamydosporia* are being used as environmentally friendly alternatives to chemical insecticides. *Metarhizium anisopliae* (Metschn.) Sorokin was one of the first organisms seriously investigated for use against agricultural pests. The pioneering immunologist Elie Metchnickoff initiated trials of this fungus against the wheat cockchafer *Anisoplia austriaca* in 1879 [1]. Products formulated with *Metarhizium* are currently used worldwide; one of the most successful biological control programmes anywhere involves treating two million hectares of sugar cane in Brazil each year with *M. anisopliae* to control spittlebugs [2]. Entomopathogenic fungi are particularly well suited for development as biopesticides because unlike bacteria and viruses that have to be ingested to cause diseases, fungi typically infect insects by direct penetration of the cuticle. These fungi are able to degrade, penetrate and assimilate the insect cuticle using a combination of cuticle-degrading enzymes and mechanical pressure, while overcoming any stresses encountered along the way [3,4]. Upon reaching the haemocoel, the fungi multiply by successfully competing for nutrients and avoiding antimicrobial proteins and circulating cells (haemocytes), which are capable of phagocytosis and encapsulation of invading microorganisms [5]. Once the host is dead, the fungus breaches the cuticle from the inside outwards, allowing the formation of conidial spores that upon dispersal start new infections. Thus, onward transmission of *Metarhizium* requires the death of the host.

Historically, a severe limitation of using *Metarhizium* and other biological control agents is that they take a long time to kill, although this is adaptive for the pathogen as it has time to maximally harvest nutrition from its host. Biotechnology can circumvent this problem, as *Metarhizium* species are experimentally very tractable and can be engineered to deliver antibodies and arthropod toxins into insects [6,7]. A *M. pingshaense* strain expressing a spider toxin has been successfully trialed against mosquito vectors of malaria in Burkina Faso [8]. The desire to use these new tools safely has driven studies on genetic containment strategies and the ‘evolvability’ of transgenic strains if they escape containment [9]. Importantly, predicting the consequences of introducing genetically modified *Metarhizium* has also thrown light on other types of human intervention such as climate change and invasive species [9].

Many of the proposed uses of *Metarhizium* have required extensive ecological studies to demonstrate efficacy and safety. Such studies are complicated as entomopathogenic fungi are very heterogeneous and occupy the same wide range of habitats as their hosts, with near ubiquity in the soil and on plants. A great deal of the biodiversity among insect pathogens has been explored at deep taxonomic levels with the genomic sequencing of *Metarhizium*, *Beauveria, Cordyceps, Hirsutella, Aschersonia* and *Ophiocordyceps* genomes [10–16] among others. These genomes have helped elucidate the genetic basis of the entomopathogenic lifestyle, by showing that convergent evolution of entomopathogenicity has occurred via the repeated evolution of an ‘entomopathogenicity toolkit’ with increased numbers of enzymes that degrade insect cuticles, and lineage-specific suites of insect-induced toxins. However, these taxa are too divergent to be useful in evaluating many important evolutionary processes which occur on a much shorter timescale. Yeast species, particularly *Saccharomyces cerevisiae*, provide the major model for rapid evolutionary processes in fungi, with some studies relating variation in isolates to adaptation to different environments [17,18]. Similar to yeasts, global distribution and comprehensive collections of isolates of *Metarhizium* species have offered a platform to decipher their ecology and evolution. The *Metarhizium* genus represents a continuum of species and strains with respect to divergence time, from recently diverged populations that are spatially restricted within continents to species that diverged more than 150 million years ago (Ma). But compared with yeast, *Metarhizium* is extraordinarily versatile and contains species that range from sexual with narrow host ranges (e.g. *Metarhizium album, Metarhizium acridum*) to plant root endophytes (colonizers of intercellular plant compartments) that are clonal with broad host ranges (e.g. *Metarhizium robertsii, Metarhizium anisopliae*) [19]. *Metarhizium* species thus provide a large number of independently evolved and experimentally tractable models of adaptation and response to diverse environments and insect hosts, illuminating the evolution and strategies of host selectivity, and reasons for the selection of sexuality or clonality. Comparative genomic studies of *Metarhizium* species that differ in metabolism, host range and root colonizing competence has revealed that proteins and gene families that alter responses to ecological interactions evolve rapidly [20], exemplifying how the accuracy of comparative analysis can be improved by relating it to different lifestyles.

To our knowledge, there has been no major review of general biological and molecular biology studies on the single genus *Metarhizium* since Roberts and St. Leger in 2004 [21]. Written at the dawn of the genomics era for entomopathogens, their focus reflected the predominance of work on *Metarhizium* as a biocontrol agent. Here, we principally focus on how recent studies on the genus *Metarhizium* have made these fungi model systems for addressing questions of wide biological significance, such as how new diseases and lifestyles originate. These studies have disentangled common themes in fungal biology from specific components involved in symbiosis and pathology, allowed broad host range pathogens to be studied in the context of narrow host range pathogens, addressed the basic question of how sexuality influences and is influenced by host specificity and habitat preference, and provided insights into the consequences of genomic changes that have accumulated during the evolutionary history of *Metarhizium*. These and other defining characteristics are retained among diverse pathogen lineages. For example, *Metarhizium* has been used in studies on the evolution of the immune system, and to provide insights into emerging human pathogens [5,22]. Furthermore, exploration of *Metarhizium* multitrophic lifestyle options is paving the way to comprehensive pest control and plant growth-promoting agents, and has identified new applications. A good example being the use of *Metarhizium* secondary metabolites (SMs) (that is low-molecular-weight molecules that are not directly necessary for growth but instead are a by-product of regular metabolism) for biotechnology and pharmaceuticals [23–25]. The prolific production of enzymes and SMs by *Metarhizium* species is linked to their broad lifestyle options, and an extremely flexible metabolism that enables them to live in various environmental conditions, with sparse nutrients [26], and in the presence of compounds lethal to other microbes [21].

## 2. The origins of the genus *Metarhizium*

**Figure 1.**
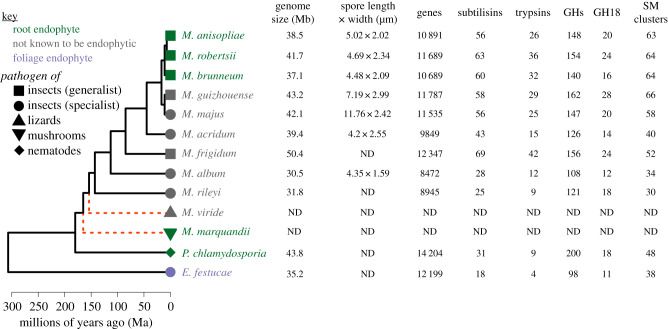
A phylogenomic tree with the estimated time of divergence for sequenced *Metarhizium* species and related fungi (solid lines). Also included (red dashed lines) are the saprophyte *Metarhizium marquandii* and the lizard pathogen *Metarhizium viride*. These have not been sequenced; their branch points on the phylogeny are estimated from a multi-gene phylogeny [32]. Right of tree, genome size, spore size and total number of genes. The number of secondary metabolite (SM) gene clusters and copy numbers of genes encoding proteases (subtilisins and trypsins) and carbohydrate-degrading glycoside hydrolases (GH), specifically GH18 (chitinases), are provided as examples of activities that likely contributed to the evolution of diverse lifestyle options. Terminal taxon names are colour coded to indicate nutritional modes (as shown in the key). Information for this figure is compiled from genome sequences described in [10,20,33–36]. Two *Pochonia chlamydosporia* genomes have been sequenced (PC170 and PC123) which differ in numbers of S8 subtilisins (31 versus 25) and GH18 chitinases (18 versus 23) [33]. We arbitrarily used the numbers for PC170.

Entomopathogenicity evolved independently in the Cordycipitaceae, Clavicipitaceae and Ophiocordycipitaceae (Sordariomycetes: Hypocreales) and these entomopathogens cluster among closely related phytopathogens, endophytes and mycoparasites consistent with repeated transitions (host switching) between plant, fungi and insect hosts [27]. The majority of species in the family Clavicipitaceae are pathogenic. The Plant-Hemiptera clade of the Clavicipitaceae contains pathogens and symbionts of plants or scale insects [28]. The other clade of Clavicipitaceae is composed of the genus *Metacordyceps* and largely comprises a radiating lineage of asexual *Metarhizium* species. including *M. anisopliae, M. robertsii, M. brunneum, M. globosum, M. acridum, M. majus, M. flavoviride, M. frigidum, M. rileyi, M. pingshaense, M. lepidiotae* and *M. guizhouense* [29]. Traditionally thought of as green-spored asexual insect pathogenic fungi, based on multi-gene phylogenies, Kepler *et al*. 2014 [29] widened the boundaries of *Metarhizium* to include fungi with non-insect hosts. These include the apparently rare and certainly little studied closely related lizard pathogens *Metarhizium viride* and *Metarhizium* (formerly *Chamaeleomyces*) *granulomatis* (the etymology of the species epithet, ‘granulomatis’ refers to the ability of the fungus to cause granulomatous disease in susceptible reptiles) [30], the saprophyte and occasional mushroom pathogen *Metarhizium* (formerly *Paecilomyces*) *marquandii* and the afore mentioned nematode pathogen *Pochonia chlamydosporia*. Because basal taxons are poorly resolved in their analysis, and ‘to limit disruption and to maintain the utility of currently recognized names', Kepler *et al*. [29] retained *Pochonia*. Since Kepler *et al*. [29], various genomic and mitochondrial DNA sequencing projects have confirmed that *Metarhizium* and *Pochonia* form a monophylectic clade [31]. Phylogenomic approaches initially placed divergence of the monophyletic *Metarhizium* lineage from clavicipitacean plant endophytes about 231 Ma [20]. A more recent tree which includes early diverged *P. chlamydosporia, M. frigidum* and *M. rileyi* places this divergence from the plant endophyte lineage *Epichloe* about 307 Ma (figure 1).

Unlike *Metarhizium* species, which principally colonize the roots of their host plants, *E*. *festucae* and other *Epichloe* species exclusively colonize intercellular spaces in plant aerial tissues, and can be vertically transmitted through host seeds or horizontally transmitted by ascospore transfer following sexual development on host inflorescences [37]. About 450 Ma the first bryophyte-like land plants probably had similar endophytic associations [38]. When roots evolved 385 Ma, mycorrhizal evolution could have progressed from these endophytic hyphae or from saprophytic soil dwellers initially attracted to root exudates. These then evolved into the vast underground fungal networks that connect trees and other plants within ecosystems, bringing nutrients and water to their roots [38]. Endomycorrhizal fungi (i.e. intracellular, penetrating into root cells) of the phylum *Glomeromycota* have remained associated with plants for more than 400 Myr [39]. A study focusing mostly on basidiomycete ectomycorrhizal fungi (surrounding plant lateral roots or penetrating between root cells), suggested that they evolved from multiple saprophytic lineages fewer than 200 Ma [40]. Basidiomycetes rapidly diversified in the Cretaceous (120 Ma), as angiosperm plants with ectomycorrhizal associations became important [38]. Insects originated at the same time as the earliest terrestrial plants about 480 Ma [41]. The root colonizing nematode pathogen *P. chlamydosporia* diverged from the core *Metarhizium* species about 180 Ma, but the oldest sequenced entomopathogen lineage, the lepidopteran specialist *M. rileyi*, only diverged 143 Ma. This is approximately 40 Ma after the appearance of lepidopterans and 30 Ma before the angiosperm radiation [42]. The root colonizing saprophyte/mushroom pathogen *M. marquandii* has not been sequenced but several multigene phylogenies place it between *P. chlamydosporia* and *M. rileyi* [29] (figure 1), consistent with the initial radiation of the *Metarhizium* lineage coinciding with the rapid diversification of root colonizing basidiomycetes and their angiosperm hosts. The assumption in the literature is that endophytism represents the ancestral lifestyle of many entomopathogens. *Epichloe* and other endophytic Hypocreales are producers of SM such as peramine that protect plants against herbivory and other fungi, and it is suggested that this may represent an intermediate stage towards the evolution of entomopathogenicity [43]. *Epichloe* uses the non-ribosomal peptide synthetase PerA to produce peramine. PerA orthologues are also encoded in the genomes of *M. rileyi* and *M. majus*, and the stalked-cup lichen fungus *Cladonia grayi*, but these are located in a seven-gene cluster that further elaborates peramine [44]. Except for PerA, these genes are absent in *Epichloe* species. Thus, the orphaned PerA gene in *Epichloe* may represent an example of reductive evolution from the ancestral seven gene repertoire, rather than acquisition of new biosynthetic capacity *en route* to entomopathogenicity. As suggested by Brundrett [38] root colonization can also progress from saprophytic soil dwellers. Strong evidence that soil dwelling root colonization is the ancestral condition for the *Metarhizium* lineage is provided by its occurrence in the earliest derived (basal) lineages such as *P. chlamydosporia* and *M. marquandii*.

## 3. The process of disease

**Figure 2.**
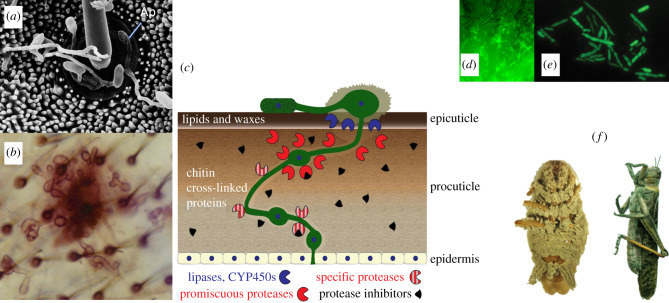
Scanning electron micrograph of *Metarhizium robertsii* strain 2575 growing on the surface of a *Manduca sexta* caterpillar (*a*). The fungus is meandering over the cuticle until it comes across hair sockets which trigger production of small terminal appressoria; hair sockets represent a zone of weakness in the cuticle which the fungus exploits [49]. (*b*) Microscope image of *M. robertsii* 2575 growing on a fly wing, incubated with pNP-propionate to demonstrate esterase activity. (*c*) Diagrammatic cross section of an infected cuticle showing a germinating *Metarhizium* spore differentiating appressoria covered in mucus, and producing an infection hyphae that grows down into and through the cuticle (green). As shown in the key, the infectious *Metarhizium* produces a sequence of enzymes during penetration starting with lipases and cytochrome P450s that target epicuticular components, and then diverse promiscuous proteases that solubilize procuticular proteins to peptides that are further broken down by more specific enzymes. The redundancy of enzymes may in part be due to protease inhibitors produced by hosts to defend the cuticle (see text). (*d*) Blastospores and short hyphal lengths of GFP-expressing *M. anisopliae* strain 549 (a generalist fungal strain that does not produce destruxins) visible in the haemocoel from outside a still living fruit fly. (*e*) A pre-mortem squash preparation of an infected fly showing blastospores and short hyphal lengths. This particular fly line has high tolerance to fungal growth; other fly lines were much less tolerant and would not contain a high fungal load before death [50]. (*f*) Images of sporulating *M. anisopliae* and *M. acridum* on cockroach and grasshopper cadavers, respectively.

Although pathogenicity evolved independently in the *Metarhizium* lineage, many factors are common to the establishment of almost every infection in spite of the diversity of pathogenic microorganisms and their hosts. The first step involves attachment to the host. In *Metarhizium*, and many other pathogens, this is initially achieved through biophysical means (electrostatic or hydrophobic interactions). Hydrophobin proteins found in a surface rodlet layer of many fungal spores mediate non-specific attachment with hydrophobic elements of the outermost cuticular wax [45,46]. Attachment is then consolidated using enzymes, mucus and specialized surface-associated adherence proteins (adhesins) [47,48]. Following attachment, *Metarhizium* species and *P*. *chlamydosporia* resemble many fungal plant pathogens in that they forcibly enter their respective insect and nematode hosts by piercing the surface with an infection peg that arises from a large ‘hold-fast’ appressorial cell that facilitates the exertion of pressure, and secretes enzymes (figure 2). The enzymes are mostly carbohydrate-degrading in the case of plant pathogens, and lipases, proteases and chitinases in the case of *Metarhizium*, reflecting the different composition of their host's integuments. In the broad host range *M. robertsii*, appressorial formation is triggered in response to the hydrophobicity and hardness of the host surface and insect-derived signals such as low levels of complex nitrogenous compounds [49,51]. Similarly, in the foliar rice pathogen *Magnaporthe oryzae*, appressorium formation is triggered by hard-surface contact and plant-derived signals such as leaf waxes and cutin [52]. In *M. robertsii, M. acridum* and the plant pathogen *Uromyces*, mechanosensitive ion channels respond to topographical information for thigmotropic growth and appressorium formation by transducing the membrane stress induced by host surface topography into an influx of ions such as Ca^2+^ [53–55]. Ca^2+^/calmodulin and cyclic AMP signalling are required for appressoria formation in *M. robertsii* after hard-surface contact primes the conidia to germinate and differentiate [56]. Ca^2+^/calmodulin signalling also triggers appressorium formation in *M. oryzae* [57]. Evidentially, *Metarhizium* species can provide a useful model for the interplay of various signalling pathways in pathogenic development.

Arthropods possess an ‘open’ body cavity (haemocoel) in which the haemolymph can flow freely over all tissues. Once inside the haemocoel, *Metarhizium* buds in a yeast-like phase (blastospores) that disperses through the insect (figure 2). Vertebrates have a closed circulatory system in which blood is contained within vessels. Nevertheless, like pathogens of insects, mammalian pathogens have to penetrate protein-rich barriers to enter their hosts and consequentially produce many proteases, and the human pathogen *Candida albicans* also has a yeast-like phase that like *Metarhizium* blastospores can survive phagocytoses [58]. Unicellular *Candida* and *Metarhizium* cells can also transform into hyphae, which are harder to phagocytose, but are capable of entering host tissues by exerting mechanical pressure.

Based on their interaction with the host, plant-pathogenic microorganisms are often divided into biotrophs and necrotrophs, although many intermediate forms exist such as hemibiotrophic pathogens which initially grow as biotrophs and later switch to a necrotrophic lifestyle [59]. Biotrophic pathogens feed on living plant cells and often show host specificity, whereas necrotrophic pathogens often show a broad host range, and rapidly cause substantial tissue damage by a combination of toxins (usually SMs) and lytic enzymes. This situation actually resembles insect infections by fungal pathogens because generalist *Metarhizium* species with a broad spectrum of hosts such as *M. robertsii* kill quickly via toxins and then grows saprophytically in the cadaver. Their toxins and enzymes frequently induce necrosis, which is to be expected from generalist pathogens that benefit from inducing cell lysis and digesting cellular components. By contrast, like many specialists, the acridid (grasshopper) specific *M. acridum* has a reduced armory of hydrolytic enzymes and other aggressive mechanisms such as toxins, and causes a systemic infection of host tissues before the host dies [10]. Many *Metarhizium* isolates may function as hemibiotrophic pathogens depending on the host genotype. Studies with the *Drosophila melanogaster* genetic reference panel (DGRP), which consists of fly lines derived from a natural population in South Carolina, showed a hidden complexity in how different, but closely related, hosts vary in their susceptibility to *M. anisopliae* so, for example, some are much better than others at tolerating *M. anisopliae* growing in their tissues before they died [50] (figure 2). Such genetic differences in host resistance and tolerance may limit the abundance and dispersal capability of the fungus, as it does with other classes of pathogen and hosts [60]. It also means that there is genetic variation in hosts available to selection and host–pathogen coevolution (Red Queen dynamics).

## 4. How do *Metarhizium* species diversify their interactions with insects: insights from genomic studies

Some *Metarhizium* kill a wide spectrum of insect hosts (generalists) whereas others are narrow host range specialists. Comparing their genomes with each other and with genomes from other entomopathogens, plant pathogens and saprophytes highlighted several features [10,20] (figure 1).

Gene content is related to *Metarhizium* genome size and linked to fungal-host ranges. As a general rule, host-restricted insect pathogens tend to have smaller more compacted genomes and fewer protein-coding genes than broad-spectrum pathogens. Genome size (number of genes) varied between 30.5 MB (8472) in the hemipteran specialist *M. album* to 50.4 (12 347) in the generalist *M. frigidum*. Genome size is also correlated with spore length (*r* = 0.51, *p* > 0.05) and particularly width (*r* = 0.88, *p* < 0.05), with *M. album* having the smallest spores (calculated from the data in electronic supplementary material table S1, Hu *et al*., [20]) (figure 1) Other features include:

(1) Both generalists and specialists have a two- to threefold higher proportion of their genome (approx. 17%) encoding secreted proteins than other ascomycete fungi, including plant pathogens.
(2) More than 50% of the species-specific genes in each *Metarhizium* species lack conserved domains suggesting many hitherto unsuspected ecological interactions involving these fungi. Most of the remaining species-specific genes have matches in the pathogen–host interaction (PHI) database or are effector-like small secreted cysteine-rich protein (SSCP) genes.
(3) Gene families associated with pathogenesis (e.g. proteases, chitinases, cytochrome P450s, polyketide synthases and non-ribosomal peptide synthetases) have expanded in *Metarhizium* species compared to saprophytes and plant pathogens, and the generalists overall have more of these enzymes than narrow host range species (figure 1).
(4) Specialists have a larger number of rapidly evolving genes (those with abundant non-synonymous mutations), compared to generalists, showing that specialization has involved rapid evolution of existing protein sequences rather than the extensive gene duplication observed in generalists.
(5) Generalists have many more transposases and orphans than specialists, and unlike specialists, show no evidence of repeat-induced point (RIP) mutations. RIP is a defence against transposons, detecting DNA duplications prior to meiosis and rendering them inactive.
(6) Specialist fungi have many fewer heterokaryon incompatibility protein (HET) domains than generalists. HETs play an important role in preserving genetic individuality through self/non-self-recognition.

### 4.1. Recognizing and responding to the presence of an insect host

The large secretomes of insect pathogens probably reflect the many microhabitats they must adapt to *in insecta*, including the cuticle and the haemolymph, as well as additional environmental habitats in the soil and with plants. These complex lifestyles are reflected in transcriptional reprogramming involving hundreds of differentially expressed genes as *Metarhizium* strains rapidly adapt to host cuticles, haemolymph or root exudate [61,62]. The ability to recognize appropriate hosts, and penetrate their cuticle, are among the necessary steps for the transition from either saprophyte or root colonizer to pathogen. After sensing an appropriate host, *Metarhizium* adapts to it by formation of infection structures and secretion of different specific effector cocktails, i.e. appressoria and a first set of effectors for the penetration stage, followed by blastospores and a different sets of organ-specific effectors for infecting insect haemolymph and tissues [62]. Many genes associated with the lifestyle switch to pathogen have been experimentally characterized. They include the previously mentioned MAD1 adhesin and hydrophobins that are responsible for adherence to the cuticle. The adhesins contain threonine-proline rich regions that mediate adhesion, and glycosylphosphatidylinositol anchor sites which localize the proteins to the plasma membrane. Expression of Mad1 allowed yeast cells to adhere to insect cuticle, while loss of Mad1 in *M. robertsii* decreased adhesion to insect cuticle, but also reduced germination and blastospore production suggesting coupling of surface sensing/adhesion to diverse downstream processes [47]. Other characterized genes include two chitin synthases for appressorial formation [63], and perilipin and cell autophagy-related proteins that regulate lipolysis, turgor pressure and formation of infection structures [64,65].

*Metarhizium* strains with very narrow host ranges exhibit less physiological adaptability than generalists and require the specific physical and chemical features of their host cuticle to stimulate infection processes [66]. Host range choices in fungi often involve G protein-coupled receptors (GPCRs) as these play essential roles in sensing environmental cues. Except for genomic comparisons, the study of GPCRs in mediating fungus–insect interactions is limited to the generalists *M. robertsii* and *Beauveria bassiana* (an insect pathogen from the ascomycete family Cordycipitaceaea), where GPCRs respond to host-related recognition signals and activate downstream pathways to control fungal differentiation and development [67–69]. Compared with the specialists *M. album* and *M. acridum*, generalists had a major expansion of GPCR-related proteins [20]. GPCR receptors are developmentally upregulated by specialists and generalists during infection processes, and the expanded repertoire in generalists may allow them to produce appressoria on multiple substrates [20].

Most of the *Metarhizium* GPCRs upregulated during insect infection [10] resemble Pth11-related GPCR genes involved in parasitism in the rice blast fungus *Magnaporthe grisea* and mycoparasitic *Trichoderma* species [70,71]. As previously mentioned, many other signal transduction genes regulating virulence in *Metarhizium* have also been implicated in pathogenicity in plant pathogens. Thus, although the host-related signals that induce germination and differentiation are different, similar signal transduction pathways may mediate these signals in very different hosts. This conservation of developmental circuitry could have been pre-adaptive for transitions between different hosts.

**Figure 3.**
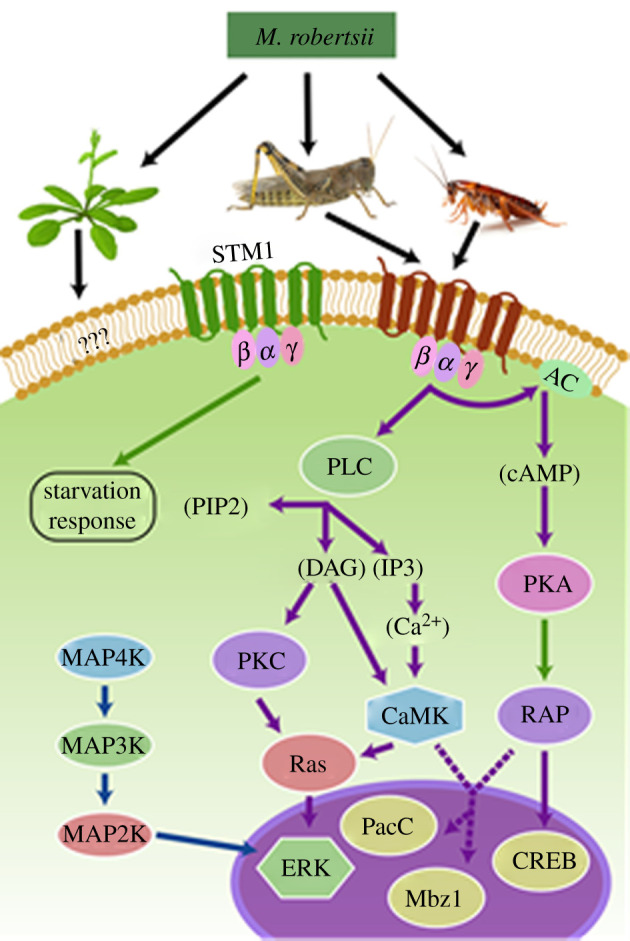
Highly simplified overview of differentially regulated signalling pathways employed by *M. robertsii* 2575 infecting cockroach and locust cuticles. Host signals are sensed by G-protein coupled and other receptors. The receptors relay signals via the mitogen-activated protein kinases (MAPK) and/or the cAMP protein kinase (PKA) relays that in turn modulate the activities of transcription factors. AC, adenylate cyclase; PLC, phosphatidyl inositol-specific phospholipase C; PIP2, phosphatidylinositol 4,5-bisphosphate; IP3, inositol 1,4,5-triphosphate; DAG, diacylglycerol; PKC, protein kinase C; CaMK, calcium/calmodulin regulated kinase; ERK, extracellular signal-regulated protein kinase; CREB, cAMP response element-binding protein.

Fungal GPCR receptors and other surface receptors mainly transmit extracellular signals to the cAMP and mitogen-activated protein kinase (MAPKs) pathways. These then amplify the signals by sequential events of phosphorylation, terminating in phosphorylation of transcription factors which adjust the transcriptional pattern of the cell to the particular condition determined by the stimulus [72–75] (figure 3). The major MAPK pathways are the Hog1-MAPK, Slt2-MAPK and Fus3-MAPK cascades. In *M. robertsii*, these recognize different host-related signals, and choreograph different subsets of extracellular cuticle-degrading enzymes and SMs during morphogenesis, invasion and diverse stresses [76]. Thus, *M. robertsii* mediates the transition from plant symbiont-to-insect pathogen through modulation of production of a membrane protein, Mr-OPY2, via alternative transcription start sites [77]. Abundant Mr-OPY2 protein initiates appressorial formation by regulating AFTF1 (appressorial formation transcription factor 1) via the Slt2-MAPK signalling pathway [77].

Studies on individual transcription factors regulated by the cAMP and MAPKs pathways are revealing many connections between seemingly disconnected strands of biochemical and molecular data. Thus, *Metarhizium* alkalinizes the proteinaceous insect cuticle by producing ammonia and it acidifies other environments by producing oxalic acid so that ambient pH can be controlled as a regulatory cue for multiple processes linked to pathogenicity [78,79]. Thus, the alkalinity of infected cuticle triggers the production of many virulence factors, including several subtilisins, trypsins and metalloproteases that are active at alkali pH, but not aspartyl proteases that are active at acidic pH [78]. Alkalization induces a zinc finger transcription factor (MrPacC) that positively controls some cuticle degrading enzymes, including chitinases even though these have a pH optima of about 5 [80]. Chitinases are only active after digestion of cuticle proteins exposes the underlying chitin to enzymolysis [81], so that linking chitinase production with alkalinization is adaptive for the fungus. At least some of the transcription factors do double duty by upregulating some genes and downregulating others. Thus, MBZ1, one of *M. robertsii*'s expanded family of 24 bZIP domain-containing transcription factors, negatively regulates subtilisins but upregulates adhesin MAD1 during germination [82], consistent with little subtilisin protease production while spores are in the process of adhering to cuticle [10].

### 4.2. Penetrating host cuticle

As a barrier, the insect cuticle prevents most fungi from being entomopathogenic. A major difference between saprophytes and entomopathogens is that the pathogens have evolved morphological (e.g. appressoria) and behavioural traits that allow them to grow down into and through the cuticle. The cuticle consists mainly of chitin microfibrils embedded in a matrix of proteins (the procuticle) and covered in lipids (the epicuticle) [3], and that is reflected in the abundance of lipid-active enzymes, proteases and chitinases in entomopathogen genomes compared with other fungi [10]. The interactions between multiple receptors and their signal transductions pathways finely tune transcription of these genes to the different sites in the host cuticle starting with the epicuticle and high-level transcription of lipases, esterases and cytochrome P450 enzymes (figure 2). The P450 monoxygenases are particularly crucial for using the long-chain hydrocarbons that predominate in many epicuticles [83]. The genome of the basal hemipteran specialist *M. album*, in particular, highlights the early expansion of proteases, as it has threefold or more trypsin genes than related plant endophytes (figure 1) and phytopathogens [20]. However, compared with *M. album* (87 proteases) and *M. acridum* (116 proteases), there has been additional expansion of proteolytic capacity in other *Metarhizium* species (average 165 proteases). The very large number of protease genes found in each *Metarhizium* genome may be due to selective pressure for the ability to produce large amounts of activity at short notice, or alternatively that a specific enzyme composition is necessary for decomposition of each fungal substrate. The second possibility is consistent with functional specialization of enzymes, thus some trypsins are ‘promiscuous proteases' and degrade -AA-AA-Arg- or –AA-AA-Lys-containing substrates (AA, various amino acids) including diverse proteins, whereas at the other extreme are enzymes that only cleave after the arginine in the sequence -Phe-Val-Arg- [84]. Similarly, analysis of the other large family of proteases, the subtilisins, revealed that one enzyme (Pr1A) was induced by nutrient deprivation, whereas other subtilisins are induced at different times as the cuticle is colonized [62]. The subtilisins differ in their secondary substrate specificities, adsorption properties to cuticle and alkaline stability [85]. Many also have amino acid substitutions that would limit the efficacy of proteases inhibitors, indicating that having large protease families may also be an adaptation to outflank expansion of host proteinase inhibitors of these enzymes [85,86]. The most promiscuous subtilisins are produced first, are usually positively charged, and they bind to negatively charged groups on the cuticle before solubilizing it. This electrostatic binding contributes to Pr1A being 33-fold more effective at solubilizing cuticle than its famous orthologue proteinase K from *Tritirachium album* [87]*.* The more specific activities are produced later, and they further degrade the solubilized cuticular proteins. These proteases are usually neutral or negatively charged which perhaps contributes to their being retained by hyphal cell walls to localize degradation products near the fungus [62,85].

Generalist *Metarhizium* species have more trypsins than any other sequenced fungus (saprophytes frequently lack them and plant pathogens have up to four) [11,20]. Some of these are produced at very high levels by infection structures before subtilisin production [10,88]. Their action also complements the subtilisins as the hydrophilic Arg-Y or Lys-Y units they cleave are on the periphery of proteins, and their action exposes hydrophobic residues susceptible to subtilisins in the protein interior [88]. To complete digestion, and provide amino acids and dipeptides for nutrition, *Metarhizium* also secretes into the cuticle numerous exo-acting aminopeptidases and carboxypeptidases, while sharply upregulating amino acid permeases [10,89–91].

Other entomopathogens besides *Metarhizium* species have more subtilisins and trypsins than plant pathogenic fungi [11]; this is consistent with niche-specific traits, i.e. traits shared by fungi that occupy the same niche irrespective of their phylogenetic position [92]. *Beauveria* is one of the best-known genera of entomopathogens and evolved into insect pathogens independently of *Metarhizium*. The expansion of proteases is dramatic in the broad host range *B. bassiana* (asexually reproducing form of *Cordyceps bassiana*) and less marked in narrow host range sexual *Cordyceps* species [13]. The relatively few genes encoding proteases in the narrow host range *Metarhizium* species and *C. militaris* suggests that their number is related to the diversity of substrates, and protease inhibitors (figure 2) likely to be encountered in multiple unrelated hosts, rather than to the efficiency with which the fungus degrades the substrate. Despite the proliferation of proteases, entomopathogenic fungi engineered to overexpress a variety of proteases, chitinases and protease–chitinase fusion proteins frequently display increased virulence [3]. This suggest that despite the very rapid expression of a large number of genes, the production of cuticle degrading enzymes by wild-type strains is sub-optimal and rate limiting in some way as otherwise overexpression should have had little effect. One potential reason for this is that although elevated activities of these proteins lead to more rapid death, they can also lead to a hyperimmune response with greater melanization of the host and reduced sporulation [93]. The rate and level of production of enzymes may therefore be traded off against increased host immunity.

Clearly, specialists lack factors that limit their ability to cause disease in multiple insects, as demonstrated by an increased host range following transfer of genes from a generalist strain to the locust specialist *M. acridum* [94]. Interestingly, the components of pathogenicity in specialists and generalists differ in the ways in which they change. The frequency of point mutations is not uniformly distributed across the genome and accumulation and fixation of non-synonymous mutations is associated with accelerated evolution. By this measure *M. acridum* and other specialists have a large number of rapidly evolving genes compared to generalists, showing that specialization has involved rapid evolution of existing protein sequences rather than the extensive gene duplication observed in generalists [20]. Several of the most rapidly evolving genes encode secreted proteins known to mediate interactions with the host, suppress host defence responses or manipulate host cell physiology. These genes are thus predicted to be primary targets of selection imposed by the host in a co-evolutionary arms race between the two interacting systems. For example, the *Metarhizium*-specific Mcl1 gene is positively selected [20]. Mcl1 is crucial for evading host immunity so its positive selection may be key to how *Metarhizium* species adapt to host defences in new hosts. Different types of transcription factors are also rapidly evolving in *Metarhizium* species. So as well as changes in gene coding regions, pathogen–host adaptation has also involved integrating molecularly changed TFs into existing gene regulatory networks to produce lineage-unique repertoires of gene expression [20]. Conversely, in generalists that are typically characterized by extensive gene duplication, a wider range of genes coding for effectors such as enzymes and toxins might make a fungus successful against a broader range of potential hosts in multiple environmental conditions. Diffuse coevolution with many insect hosts offers an explanation as to why signatures of positive selection are observed less frequently in the genomes of generalists.

### 4.3. Colonizing the haemocoel

*Metarhizium robertsii* gene products associated with colonizing the haemocoel include the RNA stabilizing cold shock protein CRP1, laccase Mlac1, an osmosensor (which signals to penetrant hyphae that they have reached the haemocoel), sterol carrier Mr-NPC2a, the collagen-like protein MCL1, enzymes for anaerobic respiration (insect haemolymph is not used for oxygen transport), and toxic SMs such as destruxins [95–101]. Some of these genes are conserved with other pathogens of different hosts, whereas others are highly adapted to the specific needs of *Metarhizium*, e.g. Mcl1 (involved in immune evasion) with its collagen domain is so far unique to *Metarhizium* and is only expressed by blastospores [95]. Mr-NPC2a is also expressed exclusively in the haemolymph; it was horizontally acquired from an insect and allows *Metarhizium* to compete with the host for growth-limiting sterols in the haemolymph [99].

Most hypocrealean insect pathogenic fungi produce large numbers of SMs, that are often synthesized by non-ribosomal peptide synthetases, polyketide synthetases and terpene cyclases. There is considerable variability in the number of SM genes among *Metarhizium* species, but as a general rule generalist *Metarhizium* species., such as *M. robertsii*, possess a greater potential for the production of SMs than specialist strains and other ascomycetes [10,20,98,102] (figure 1). Thus, the available *Metarhizium* genomes contain between 10 to 20 Pks (polyketide synthase) genes, and the most notable lineage-specific expansions occur in generalists [20]. Some of the compounds produced by these SM gene clusters have been identified, such as destruxins (dtxs) (cyclic hexadepsipeptides) produced by broad host range *Metarhizium* only [102]. A characteristic of generalist *Metarhizium* strains is that they have experienced extensive gene duplications. An important principal of molecular evolution is that following a duplication event, one gene copy can keep the original function while the other ‘spare’ copy can acquire new functions. Two of the Pks gene clusters in most *Metarhizium* species were formed from the duplication of an ancestral Pks gene cluster after *M. acridum* split from *M. album*. Subsequent diversification of the sequence resulted in Pks2 which is involved in the formation of infection structures, while Pks1 is required for conidial pigments that increase tolerance to UV and heat [103].

### 4.4. The relationship between genome size and lifestyle options

The different lifestyle options and broad host range of generalists may require increased gene content and more complicated regulation in response to a wider need for cuticle degradation, detoxification and toxin biosynthesis for various host types. Such factors are likely to result in an increased genome size, which in turn requires a higher nutritional intake to meet DNA biosynthesis and the production of spores. Like plant biotrophs, specialized *Metarhizium* species tend to conform with the usual evolutionary trend of parasitic genome reduction, although the size of the *M. album* genome (30.5 Mb) is not much smaller than that reported for the average for Ascomycota (36.9 Mb) [104]. Specialized *Metarhizium* species may still have a saprophytic existence in soil the requirements of which may limit genome reduction. *Ophiocordyceps sinensis* (used in traditional Chinese medicines and also known as ‘Himalayan Viagra’) provides an interesting exception to the usual evolutionary trend of parasitic genome reduction, and an example of extreme specialization. It is special in the way that it infects its early instar ghost moth (*Thitarodes* species) caterpillar hosts through spiracles or the mouth, and thus avoids the cuticle degradation step, and then lives quietly in the host for several years before killing it [105]. *Ophiocordyceps sinensis* has a small gene content, and a much smaller number of CYP52 enzymes, subtilisins, trypsins, chitinases and aspartyl proteases than *B. bassiana, C. militaris* or *M. robertsii*. Despite this, *O. sinensis* has a hugely inflated genome mediated by the accumulation of repetitive elements [72]. *Ophiocordyceps sinensis* is dependent on the host, and will rarely if ever need to spread quickly, relaxing the trade-off of genetic toolkit versus genome size. Evidently, differences found among the insect pathogens in protein family size are related to their *modus operandi* and host range.

The relationship between genome size and spore width suggest that genome size may influence selectable ecological and morphological traits such as spore counts, spore mass and manner of dispersal. Lavergne *et al*. [106] reported that genome size reduction can trigger rapid phenotypic evolution in invasive plants, and that phenotypic effects resulting from smaller genome sizes increased invasive potential. However, a large majority of the USDA ARSEF collection of *Metarhizium* strains are from generalist species, and in contrast to most generalist species, specialists often have localized distribution, indicating that being a generalist is a successful ecological strategy for *Metarhizium*, perhaps because it is usually linked with plant associations. However, with increased interest in the genus, new *Metarhizium* species are being reported yearly. Many of these are rare locally distributed specialists. Thus, a 2020 paper [107] describes 19 new species with narrow host ranges in Thailand, seven of which produce a sexual morph.

## 5. A mosaic of ecological interactions; not just entomopathogens

Like many of the ‘original’ *Metarhizium* species, *M. marquandii* and *P. chlamydosporia* are common soil-borne fungi distributed throughout temperate to tropical latitudes worldwide including forests and grassland. However, a distinguishing characteristic of both *M. marquandii* and *P. chlamydosporia* is that they routinely produce asexual thick-walled resting chlamydospores in harsh conditions. There are few reports of chlamydospore production by more recently diverged *Metarhizium* species [108]. Our understanding of the biology and genetic basis for formation of chlamydospores is poor. Perhaps entomopathogenic fungi can escape some harsh environments and disperse in their hosts reducing the need for chlamydospores, but it would be useful to learn how to induce their proliferation as chlamydospores have many advantages as a biocontrol agent, including resistance to environmental stress and a longer shelf life.

**Figure 4.**
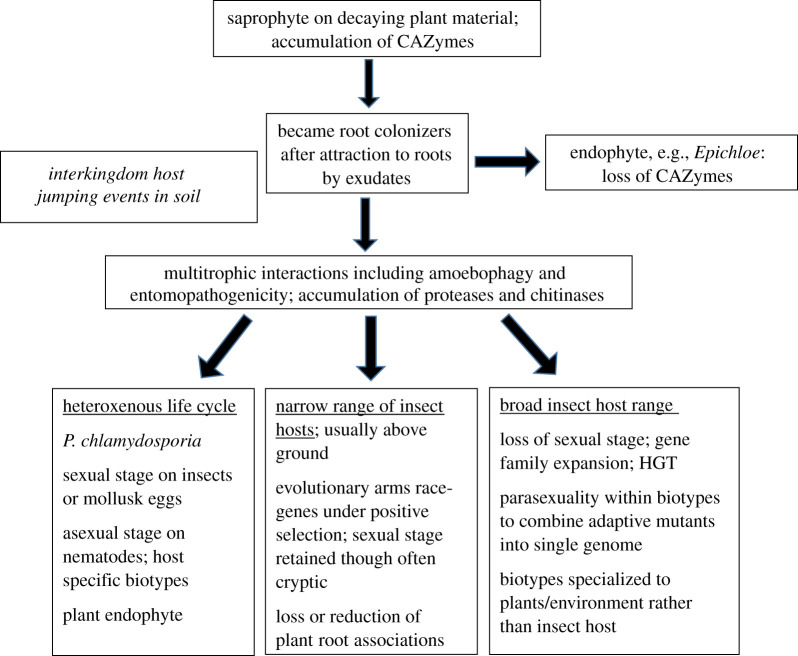
Major transitions in the evolution of *Metarhizium* species. The lineage may have arisen from saprophytes that accumulated carbohydrate degrading enzymes (CAZymes) to degrade plant material, and presumably first became endophytes after attraction to roots by exudates. The rhizosphere provides a habitat where amoeba, nematodes, insects and fungi interact facilitating interkingdom host jumping. The close relationship of *Metarhizium* and *P. chlamydosporia* may indicate that parasitism evolved in a common ancestor. Entomopathogenicity may have evolved first, assuming asexual *P. chlamydosporia* evolved from sexual forms, as sexual morphs are too large for nematodes, but have been found on beetle larvae. *Metarhizium* genotypes with broad host ranges have been selected principally to soil and plant root habitat, not to host insect. Their population structures are clonal with parasexuality within each biotype potentially combining adaptive mutations that arise in separate lineages into one genome. The absence of sex allows extensive gene duplication that together with horizontal gene transfer (HGT) has provided generalists with a large armamentarium of enzymes and toxins able to overcome many insects. Generalist *Metarhizium* species and *P. chlamydosporia* have retained the ancestral root association, but compared to saprophytes, *Metarhizium* species at least have an additional ability to pass animal-derived nitrogen to the plants in exchange for carbon. Many other genotypes with evolutionary histories of insect host specificity have retained sexuality and have a larger number of rapidly evolving genes, possibly as part of an evolutionary arms race with hosts. For the most part these fungi have specialized to above-ground insects, and have reduced plant associations.

*Pochonia chlamydosporia* is a fungal egg parasite of root-knot and cyst nematodes able to colonize the roots of several plant species and induce defence mechanisms in some plants against root-knot nematodes [109]. Variability in response may reflect the degree to which isolates colonize the rhizoplane of a particular plant, but even in the absence of nematodes *P. chlamydosporia* promotes the growth of tomato and lettuce [110]. Genetically distinct variants of *P. chlamydosporia* are associated with different host nematode species. It is predicted that these variants will eventually be elevated to species rank, and that many more species will be identified [111,112]. That would follow the precedent set by *M. anisopliae* which was originally a complex of multiple varieties that were only recognized as species in 2009 [113]. Soil samples taken around coffee plants showed that *Metarhizium* and *P. chlamydosporia* isolates predominate in different plots at different times [112], presumably related to host densities as counts of *P. chlamydosporia* in the rhizosphere increase with the nematode population in the roots [114]. It is conceivable that more intensive and focused sampling will reveal that *P. chlamydosporia* diversification on nematodes, and perhaps plants, parallels in richness *Metarhizium* species divergence on insects. However, without parallel in *Metarhizium*, two *P. chlamydosporia* varieties, namely var. *chlamydosporia* and var. *catenulate*, produce sexual morphs on alternate hosts: molluscs (snail eggs) and insects (beetle larvae), respectively. This is believed to be because nematodes are too small to support the robust size and complexity of the sexual structure [112]. Assuming asexual forms evolve from sexual forms, pathogenicity to nematodes presumably derived from an ancestor that had a bigger host, and pathogenicity to insects (or molluscs) may be the ancestral condition (figure 4).

The genomes of several nematophagous fungi are available [115], including *P. chlamydosporia* [33,116] and they usually contain gene expansions in families of chitin-degrading enzymes and subtilisin proteases similar to those found in entomopathogens. These are probably adaptations to digest the protein/chitin nematode eggshell but also likely facilitate interconversion between these different lifestyles. It is noticeable that the gain of subtilisin genes in *P. chlamydosporia*, relative to *Epichloe* (figure 1) and plant pathogens (e.g. the 10 in the related *Fusarium graminearum* [10]), preceded (and therefore could not have been caused by) the evolution of entomopathogenicity, unless *P. chlamydosporia* itself had an entomopathogenic ancestor as hinted by the beetle host of the var *catenulate* sexual morph (figure 4). Since these pathogens and their plant root, nematode and insect hosts are all soil dwelling, ecological barriers for host switching are probably smaller than for other parasitic vector systems. Furthermore, *Metarhizium* species including *P. chlamydosporia* possess a well-developed carbohydrate-degrading metabolism, including many traditional plant pathogenesis-related genes, consistent with ongoing interactions with plants. Many plant pathogens need glycoside hydrolases (GHs), pectate lyases, and cutinases to degrade the plant cuticle (waxy layer) and cell wall. The number of GHs possessed by *B. bassiana* (145) [11] *M. robertsii* (154), *M. acridum* (126) and *P. chlamydosporia* (200) is more than the endophytic close relation of *Metarhizium*, *E. festucae* (98), but only *P. chlamydosporia* is a match for plant pathogens (average 200) [11]. This is because overall insect pathogens have fewer genes associated with plant colonization and utilization than plant pathogens. This includes fewer oxidative lignin enzymes (average of 29 in insect pathogens versus 40 in plant pathogens), carbohydrate esterases (9 versus 33), cutinases (4 versus 12) and pectin lyases (8 versus 20) [11]. However, *P. chlamydosporia* and generalist *Metarhizium* species show profiles of carbohydrate-active enzymes (CAZymes) that are more similar to plant pathogens than to other insect pathogens. In general, broad host range *Metarhizium* species contain more CAZymes than symbiotic and biotrophic fungi, which depend on living plant tissues for their nutrition, and fewer than most necrotrophs that use dead plant material [117]. The basidiomycete *Laccaria bicolor* was used by Zhao *et al*. [117] as an exemplar of a plant root symbiont that contains a small number of CAZymes. As intercellular endophytes that usually colonize aerial parts of plants, *B. bassiana* and *E. festucae* presumably possess mechanisms to avoid stimulating plant defences. Fungal xylanases are known to trigger plant immune responses, and unlike *Metarhizium* species, *E. festucae* and *B. bassiana* lack GH11 xylanases [11,105]. Presumably, the last common ancestor of *E. festucae* and the *Metarhizium* clade had the full repertoire of CAZymes retained in current *Metarhizium* species, and some of these have subsequently been lost in the *E. festucae* lineage. Along with the PerA gene cluster, these represent examples of reductive evolution which suggest that the common ancestor may not have had the same systemic endophytic lifestyle currently engaged in by *E. festucae.* Possibly, the *E. festucae* lineage, as well as *Metarhizium* species, are descended from soil colonizers resembling the opportunistic saprophytic root colonizing *M. marquandi* rather than foliage endophytes.

This pattern of gain and loss of CAZymes is a feature of the *Metarhizium* clade. Of the sequenced species, *P. chlamydosporia* has the most CAZymes [118] (figure 1), and therefore can likely metabolize the greatest diversity and complexity of plant substrates. Thorough degradation of cellulose requires the collaboration of endoglucanase, cellobiohydrolase and β-1, 4-glucosidase, and all strains retain many of these enzymes. However, only *P. chlamydosporia* retains an AA9 lytic polysaccharide monooxygenase (LPMO), and GH6 and GH7 cellobiohydrolases that degrade crystalline cellulose, presumably facilitating plant cell wall decomposition. This may explain why *P. chlamydosporia*, unlike endophytic *Metarhizium* strains, frequently penetrates into plant cells forming hyphal loops for nutrient absorption [119]. *Metarhizium* strains grow between cells, which would be facilitated by pectinases that loosen the cell wall, and generalist *Metarhizium* strains are well endowed with these. *Metarhizium rileyi* and *M. album* have the fewest CAZymes (figure 1) suggesting that some additional plant degrading enzyme activities became superfluous in these specialized lineages. This is in line with the narrow host-range associated environment being more consistent and the broad host range associated environment, particularly the soil habitat, being more diverse. Among GHs, GH18 (chitinases), which play an important role in hydrolysing the chitin-rich insect cuticle, were not distributed in the same way as other GHs, with generalist *Metarhizium* strains having a few more than *P. chlamydosporia* (figure 1)*.* Overall, chitinases are overrepresented in *Metarhizium* (24 in *M. robertsii* and 14 in *M. acridum*; 5–14 in plant pathogens) [10]. Other entomopathogens such as *B. bassiania/C. militaris*, and mycoparasites like *Trichoderma* species also have expanded repertoires of chitinases, but phylogenetic analysis revealed that most of the responsible gene duplication events have occurred since *B. bassiania/C. militaris*, *Metarhizium* and *Trichoderma* species diverged from a common ancestor, suggesting their abundance in each clade is due to convergent evolution [11].

Several insect pathogenic *Metarhizium* species can develop symbiotic associations with plant roots. *Metarhizium robertsii* is attracted by chemical signals, particularly raffinose, released by plant roots [120]. The initial steps of symbiosis establishment involve adhesion via an adhesin (MAD2) and growth over the root surface, particularly the mucilage secreting root tip. Colonization includes intercellular growth in the outer root layers. It is not known how *Metarhizium* species penetrate the plant root epidermis, but intercellular hyphae grow in a step-wise fashion around the cell walls [120]. *Pochonia chlamydosporia* penetrates the root epidermal cell walls using appressoria as it does nematode eggs [121]. The fact that as well as having MAD1 for insect cuticle, *M. robertsii* upregulates a specific plant adhesin (MAD2) in the presence of plants demonstrates that it has specialist genes for a bi-functional lifestyle [47]. Other specifically regulated genes include a novel oligosaccharide transporter for root-derived nutrients required to colonize the rhizosphere and roots [120], an RNA binding protein that has important roles in both saprotrophy and pathogenicity [96], and an invertase that aids in the regulation of hydrolytic enzymes and provides a plant-derived signal restricting fungal growth [122]. Although an isolate of *P. chlamydosporia* was a better plant root colonizer than *M. robertsii, M. acridum, M. flavoviride, M. brunneum* and *M. pingshaense* [123], a rhizosphere competent, avirulent mutant of *M. robertsii* survived better in grassland soil than an insect pathogenic mutant unable to adhere to root surfaces, demonstrating the importance of plant roots in maintaining populations of *M. robertsii* [19]. *Metarhizium robertsii* also persisted at high levels on cabbage roots throughout the winter [124] so another advantage of being a root endophyte is that it provides saprophytic access to nutrients after the death of the plant. Not included in the survey [123], *M. marquandii* has been known for a long time to associate with plant roots and to germinate in their presence [125]. *Metarhizium marquandii* has not been sequenced, but it is known to thrive on a very wide range of organic substrates, including sewage, caves, sand dunes and various soils [125], and it occasionally parasitizes the edible mushroom, *Cuphophyllus virgineus* colouring its lamellae violet [126]. Given its abundance, if *M. marquandii* did exhibit more than low virulence towards a broader array of living hosts that would likely have been noted. There is a report that both *P. chlamydosporia* and *M. marquandii* can kill soft bodied insects (white fly *Bemisia tabaci*) in the laboratory, and sporulate on the cadavers [127]. Sporulation is an important consideration as it is necessary for transmission to a new host. The sexual stage of *P. chlamydosporia* var. *catenulata* is produced on beetle larvae [112], although another isolate of *P. chlamydosporia* was not entomopathogenic [128].

A comparative genome analysis of seven entomopathogen *Metarhizium* genomes [20] placed the hemipteran-specific *M. album* as basal in the *Metarhizium* clade with an estimated divergence time about 117 Ma [20]. It was suggested that the close physical proximity of a plant-associated ancestor of *M. album* to plant-sap sucking hemipteran bugs may have facilitated this particular host switch to insects [20]. However, the genome of *Metarhizium* (formerly *Nomuraea*) *rileyi* suggests that this species diverged before *M. album* (figure 1)*. Metarhizium rileyi* causes large epizootic events almost exclusively in noctuid lepidopteran species [129]. *Metarhizium rileyi* overwinters in soil and work in the 1970s suggested that soil-borne contamination of seedlings provides an inoculum [130]. In the light of modern knowledge, it would be interesting to check whether *M. rileyi* is endophytic in foliage, as this might facilitate infection of foliage-eating caterpillars. *Metarhizium rileyi* is not known to colonize plant roots, and its preferred hosts living on foliage rather than soil may mean that root associations are not adaptive. Likewise, *M. album* and *M. acridum* are also specialists of foliage pests (hemipterans and acridids, respectively), although *M. acridum* can form weak endophytic and rhizospheric interactions in the laboratory [128]. Overall, these results suggest a link between specialization and switching to a foliage dwelling host, although with possible retention of some ancestral endophytic capacity*. Metarhizium majus* is an exception in that it is most frequently observed as a pathogen of larval rhinoceros beetles in rotting wood, and unlike other specialists has been found in soil in nature [131]. One *M. majus* strain was not rhizosphere competent in a laboratory study [132], but this does not of course rule out specialization to a narrow range of plants in the field that were not represented in the laboratory. *Metarhizium album* and other specialist *Metarhizium* species retain some enzymes devoted to degradation of plant materials, as does *O. sinensis* which may have a plant endophytic stage to facilitate infection of root-eating host larvae [16,20,133]. *Ophiocordyceps sinensis* may not need many CAZymes if it exploits damage caused by the herbivory of its hosts to enter plants. However, the amphibian pathogen *Batrachochytrium dendrobatidis* is one of many examples of a pathogen with lignocellulases that has no known association with plants [117]. These CAZymes may be relics inherited from an ancestor that associated with plants or be used in a current unknown association or saprophytic phase.

## 6. How do plants benefit from their interactions with *Metarhizium* species?

Many studies have shown that entomopathogenic fungi, particularly *B. bassiana* and generalist *Metarhizium* species, can increase plant growth, particularly under stress. Examples with *Metarhizium* species include a significant increase in onion yields [134], and bigger tomato plants [135], as well as increased growth of soya bean seedlings during salt stress [136] and maize plants that did not receive fertilizer [19]. In most of these studies, *Metarhizium* was applied as a soil inoculation or seed treatment. In general, fungal associates typically boost plant growth by absorbing phosphorus and other nutrients, capture nitrogen from decaying organic matter, and help store carbon in the soil [137]. In addition to suppression of herbivores via nematophagous or entomophagous activities, *Metarhizium* species have multiple such direct growth-promoting effects on plants. These are still being elucidated but include solubilizing rock phosphorus in soil making it more accessible to plants [138], and transfer of nitrogen by hyphae connecting insect cadavers and plant roots [139]. As quid pro quo, *M. robertsii* exchanges this insect-derived nitrogen [139] for ‘photosynthesate’ i.e. carbohydrates [140]. Moonjely *et al*. [141] suggest that this exchange was the driving force behind evolution of the plant–*Metarhizium* partnership: as conduits of insect-derived nitrogen, these fungi become an indispensable partner underground. The benefits to the plant will be conditional on soil fertility [19], but mineral nutrients (especially phosphorus and nitrogen) are among the most important limiting factors for plant growth in natural ecosystems [38]. Nitrogen is also an ancestral need; the first mycorrhizas were likely formed by a *Geosiphon*-like fungus that could tap into an abundant supply of nitrogen obtained from associated cyanobacteria [142]. A fungus colonizing an insect presumably has nitrogen in excess of its immediate requirements, and it would clearly increase opportunities for nutrition if the colonizing endophyte could exploit diverse insects, which potentially could select for a broad host range. Thus, the association with roots combined with some combination of nematophagous, mycoparasitic or entomopathogenic characteristics might have evolved as part of a symbiotic relationship in which the fungus feeds the plant as well as protects it against parasites and herbivores. These additional pathogenic lifestyles would be advantageous to the fungus if they enable it to escape competition, predation and parasitism from other soil organisms, and build up population levels greater than the carrying capacity of the plant root and rhizosphere. The ubiquity of generalist *Metarhizium* and their long history as plant root colonizing insect pathogens is evidence that the benefits of exchanging photosynthates for nitrogen have outweighed the costs.

**Figure 5.**
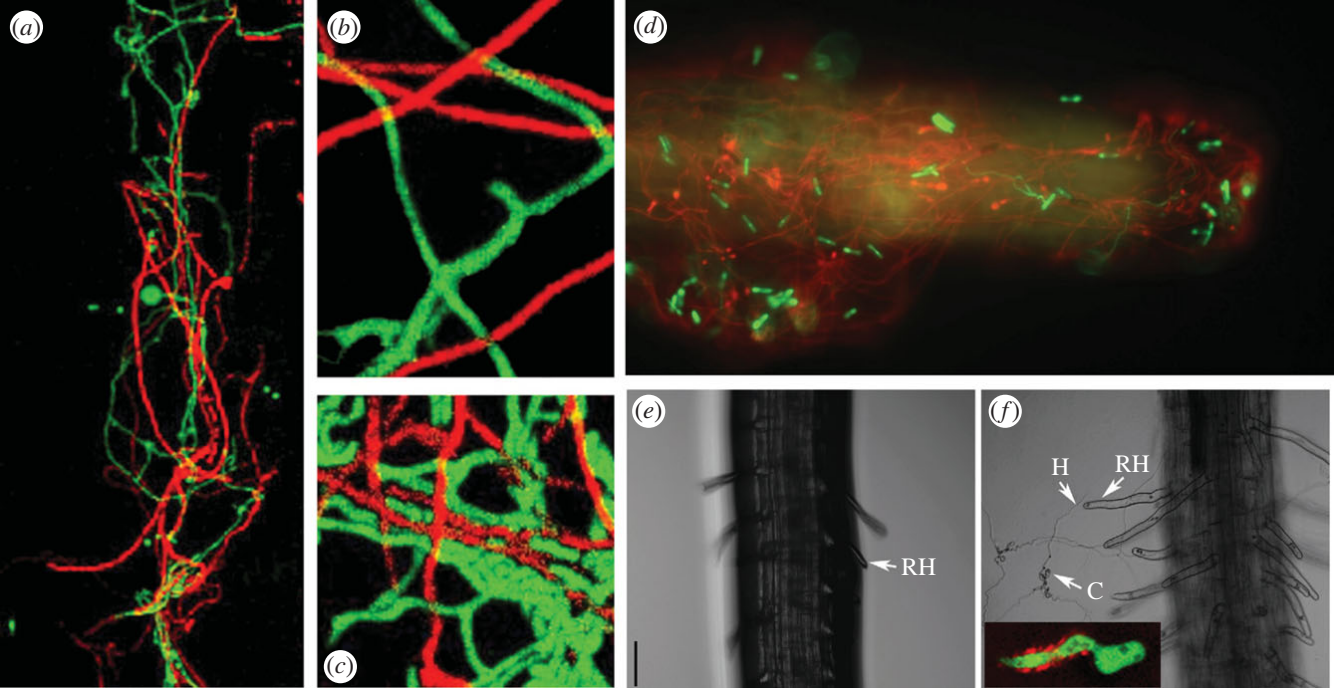
*Metarhizium robertsii* strain 2575 expressing RFP and *Trichoderma harzianum* strain T12 expressing GFP growing down a grass root (*a*) or against a plastic surface in the presence of 0.01% (*b*) or 0.1% (*c*) yeast extract medium. (*d*). *Metarhizium robertsii* strain 2575 expressing RFP and *M. majus* strain 1946 expressing GFP co-inoculated onto *Arabidopsis* roots; strain 2575 forms a network over the root whereas 1946 shows low level germination. (*e*) Effects of *M. robertsii* inoculation on *Arabidopsis* root hair development. *Arabidopsis* was grown for 10 days on agar medium plus (+Mr2575) or minus (−Mr2575, control). RH, root hair; C, spore; H, hypha. Scale bar, 30 µm. The inset shows immunolocalization of auxin IAA (red) in the mucus secreted by a green fluorescent protein-tagged germinating *M. robertsii* 2575 spore (frames E and F from reference [132]).

As befits an ancient association, there is evidence that sophisticated and subtle signalling underlie plant–*Metarhizium* interactions. *Beauveria bassiana* and several *Metarhizium* species including *M. marquandii* produce the phytohormone IAA (indole acetic acid), the best-known auxin, through Trp-dependent pathways [132,138] (figure 5). IAA is involved in tropism responses, cell division, vascular tissue differentiation and the initiation of root formation [143], and its synthesis by *Metarhizium* species modifies the root architecture, increasing the root area for colonization by *Metarhizium* and boosting uptake of nutrients by the plant [132] (figure 5). Interestingly, *M. robertsii* also produces IAA on insect cuticles where it is required for maturation of infection structures, and in insect haemocoel where it exerts a toxic effect, possibly activating the prophenoloxidase cascade and inducing the production of reactive oxygen species [132]. Plants also use indole alkaloids to resist insects [144], and synthesized indole derivatives are highly toxic to insects [145]. Entomopathogenic *Metarhizium* species probably evolved from basal *Metarhizium* species resembling *M. marquandii* that also endophytically colonize plant roots, and some genes for insect pathogenesis may have been co-opted from genes involved in endophytic colonization [146]. Genes for auxin biosynthesis may be among those involved in lifestyle transitions towards insect pathogenicity.

*Metarhizium* also seems to modulate plant production of hormones, although how it elicits these changes is unknown. Thus, peanut roots colonized by *M. anisopliae* have lower levels of the immune regulating hormone salicylic acid consistent with suppression of plant defence, as well as physiological changes in oxidation-reduction, transport and metabolism that show the plant is acutely aware of the fungus [147]. However, the peanuts transcriptional response to *M. anisopliae* was quite different than its response to a related plant pathogen, *Fusarium oxysporum*, which rapidly induced a plethora of defence responses [147]. *Pochonia chlamydosporia* reduces the colonisation, root damage and associated stress in wheat roots caused by the fungal root pathogen *Gaeumannomyces graminis* probably in part by priming the plant immune responses [148], while induction of genes involved in stress response (e.g. heat-shock proteins), hormones and plant immunity were enriched in barley roots colonized by *P. chlamydosporia* [149]. *Pochonia chlamydosporia* can also benefit plants via the jasmonate signalling pathway by stimulating plant growth, reducing flowering time and increasing seed production [150]. It is likely that at least some of these findings from a single pathogen–host system will not apply to other fungus–plant associations, as these fungi have diverse roles in inducing plant growth, and reducing or enhancing defences, that depend upon the plant species as well as the fungus [19].

The reasons for this variation are unclear, although *Metarhizium* strains produce different amounts of IAA [132], and vary in the extent of their interactions with plants [19]. Variation is consistent with ecological specialization, with broad host range *Metarhizium* species being associated with particular environments rather than insect hosts, and so they have more precise requirements for the habitat and plant they colonize than they do for the insects they infect. As a rule, generalist *Metarhizium* are not subtle regarding their insect hosts, but instead ‘loaded for bear’ with a large armamentarium of enzymes and SMs able to kill many insects. Conversely, evidence for coevolution with plants is that *M. robertsii* preferentially associates with the roots of grasses, *M. brunneum* with shrubs and *M. guizhouense* with trees [151,152]. By contrast, the specialist *M. acridum* that targets locusts and related acridids diverged 48 Ma when grass-feeding acridids first appeared, implying host-driven divergence [20]. Aside the flora in a particular habitat, the distribution of different genotypes of generalist *Metarhizium* will likely depend upon environmental factors, such as temperature and humidity, and soil conditions, such as pH and organic matter content [153]. *Metarhizium robertsii* has mechanisms for rapidly evolving to new soil habitats which involve changes in expression of cell wall and stress response genes but not virulence genes [9].

## 7. *Metarhizium*–microbe interactions

As shown by their antagonism to plant pathogenic fungi such as *Fusarium* [154], ability to survive exposure to diverse toxic chemicals including heavy metals [155], and pathogenicity to soil amoebae [156], at least some *Metarhizium* isolates have considerable additional flexibility in their trophic capabilities besides plants and insects. The relationship between fungi and amoeboid protozoans is largely unexplored, although as both groups thrived in early land systems long before land plants and terrestrial arthropods, they presumably played a part in each other's ancestral diversification [157]. Amoeboid protozoans are able to predate fungi but *M. robertsii* can survive phagocytoses by the soil amoeba *Acanthamoeba castellanii*, following which it upregulates the glyoxylate cycle using intracellular lipids to grow within and then escape from these organisms [158]. This led to the suggestion that the ability of *Metarhizium* to survive and escape phagocytosis *in insecta* may have arisen as a result of adaptations necessary for survival in interactions with soil protozoa or other soil organisms [158]. It has also been suggested that this may be a general principal, and a wide variety of pathogenic and potential future pathogenic fungi may be under selection by predatory soil organisms [159]. Given that *Metarhizium* and amoeboid protozoans are both very common inhabitants of soils, their so far unexplored ecological interactions are likely to be frequent, and clearly deserve study. The same holds true for prokaryotic inhabitants of *Metarhizium* environments. The potential interactions between entomopathogenic fungi and microbes in soil, plants and insects are poorly characterized, but some insect pathogens appear able to suppress host microbes while conversely some insects have acquired microbes that produce antifungal compounds [160]. Konrad *et al.* [161] demonstrated that infection with the obligate insect ectoparasitic fungus *Laboulbenia formicarum* protected its ant hosts from *M*. *brunneum,* perhaps due to stimulation of the immune system or an intensification of grooming behaviours.

Typically, laboratory studies deploy a single fungal interactor with a plant, whereas in nature *Metarhizium* will be interacting with a whole plant-associated microbiome [162] These will include other beneficial microbes such as growth-promoting rhizobacteria or fungal biocontrol agents including *Trichoderma* species (figure 5). SMs such as 6-pentyl-alpha-pyrone, peptaibols, harzianum A and aspinolides produced by certain *Trichoderma* species act as elicitors of plant defence against pathogens and often also show positive effects on plant growth and development [163]. It would be surprising if some of the unknown *Metarhizium* SMs did not have functions contributing to the impact of this fungus on plants. Comparative genomics reveals *Metarhizium* and *Trichoderma* are related [10], and there are commonalities between them in how they interact with plants. Thus, *M. robertsii* and the rhizosphere-competent biocontrol fungi *Trichoderma virens* both employ invertase to metabolize sucrose and control root colonization [122,164]. However, mechanistic differences in root colonization suggest that *Metarhizium* and *Trichoderma* have independently evolved rhizosphere competence [122]. For instance, *Trichoderma* uses a hydrophobin to adhere to roots [165], whereas *Metarhizium* uses an adhesin (Mad2) [47] although it also expresses a hydrophobin of unknown function in response to root exudates [165]. It is likely that many fungi have independntly evolved their own multi-faceted and robust mechanisms to overcome the challenges encountered on plant roots. Strategic differences may even extend to different strains of *M. robertsii*, as some produce highly conidiating colonies on soil insects (sleepers) whereas others produce more hyphae and fewer conidia (creepers) [166]. It was suggested that sleeper strains wait until a host insect or plant encounter the conidia whereas creepers proactively search through the soil for a protagonist.

*Trichoderma harzianum* is one of the most abundant root colonizers in agricultural fields, and the most metabolically diverse of the *Trichoderma* species, credited with numerous beneficial effects on plants [167,168]. The shared habitat of *T. harzianum* and *Metarhizium* species could potentially lead to competition for resources, and both *Metarhizium* [169] and *Trichoderma* [170] have activity against plant pathogens, with *Trichoderma* species being specialized mycoparasites [147]. Despite this at least some biocontrol isolates of *M. robertsii* and *T. harzianum* show no overt hostility to each other (figure 5), which could facilitate production of consistent biopesticide products based on combinations of microbes that economically achieve breadth of action. Compared to *Trichoderma,* some broad host range root colonizing *Metarhizium* species germinate in response to very low levels of plant root exudates, which suggests the two fungi may not occupy precisely the same niche in nature [171], with *Metarhizium* being adapted to earlier access to exudates. Some *M. robertsii* strains, including strain 2575, are particularly hypersensitive to root exudates and use a raffinose transporter (Mrt) to produce chemotropic growth towards roots [120]. Although not included in the study that defined ‘sleepers’ and ‘creepers’ [166], *M. robertsii* 2575 sporulates heavily on most insect cadavers consistent with it being a ‘sleeper’, but senses, germinates and ‘creeps’ towards plant roots in its vicinity, suggesting a mixed strategy that conserves energy to when it would be most propitiously expended. *Metarhizium robertsii* 2575 also germinates with the very low nutrient levels on beetle cuticles (it was originally isolated from a weevil) [172], so its responsiveness to sparse nutrients is a feature of its adaptation to multiple lifestyles.

The multiple associations of at least some *Metarhizium* species with insects, plants, amoeba, and maybe other organisms, probably accounts for them being among the most abundant fungi isolated from soils, with titres reaching 10^6^ conidia per gram in grasslands [173]. Their abundance and ubiquity suggests that they are transferring nitrogen from insects to plants on a very large scale creating an additional branch of the soil nitrogen cycle [139]. However, the links between fungi and other ecosystem components are rarely this clearly visible. For example, *M. marquandii* and other *Metarhizium* species have been isolated from marine environments [174,175] but whether they are terrestrial fungi accidentally washed into the sea or have an unexplored marine ecology is unknown. There is a report of *M. anisopliae* in the coelom of a sea sponge [175], which raises the possibility that these fungi might also associate with marine animals.

## 8. Traits for being multitalented

Tolerance to diverse toxic chemicals may be a prerequisite for the diverse ecological roles of *Metarhizium* species. Consistent with this, the entomopathogen *Isaria fumosorosea* is inhibited by chemical plant defences and does not form complex associations with plants [176,177]. High concentrations of root exudate also inhibit growth of specialist and generalist strains of *Metarhizium* to varying extents, indicating that strains respond differently to the inductive and repressive components of root exudate [171]. A non-root colonizing fungus, *Aspergillus niger*, was highly sensitive to the inhibitors in root exudate, indicating that it is not adapted to resist antimicrobial exudate components [171]. Similarly, insect cuticles have surface antifungal chemical defences such as short-chain fatty acids and other small molecular toxins (including peptides) [3]. Fungi capable of rhizosphere competence or entomopathogenicity have presumably evolved some degree of resistance to these compounds. A feature of *Metarhizium* species, including the non-entomopathogenic *M. marquandii,* is that they are often found in strongly metal polluted areas [178,179]. Indeed, *B. bassiana* (another endophytic insect pathogen) and *Metarhizium anisopliae* can be used as efficient biosorbents for Pb(II) and Cd(II) from aqueous metal solutions [180]. Metals clearly play an important role in *Metarhizium* biology as shown by the large number and diversification of detoxification systems in *Metarhizium* genomes [10]. It is unlikely to be a coincidence that *M. anisopliae* and *B. bassiana* share their tolerance to metals with mycorrhizal fungi [181]. *Beauveria bassiana* mostly colonizes aerial parts of the plant, but *Metarhizium* needs to compete for iron with other members of the plant root microbiome, and tolerance to metals could add additional benefit to *Metarhizium* symbiotic associations with plants. Farias *et al.* [182] found that a consortium of fungal isolates (*M. anisopliae*, *P. chlamydosporia*, *B. bassiana*, *Purpureocillium lilacinum* and *Trichoderma asperella*) improved plant tolerance to heavy metals and boosted growth.

Metals are also known to play a large role in fungal infection processes of vertebrate and plant hosts [183]. Because iron sequestration is crucial for pathogenic microorganisms, these hosts have developed iron-withholding defence mechanisms in a process aptly named ‘nutritional immunity’. A role for nutritional immunity in insect defences is evidenced by the fact that a functional ferricrocin (that is functions to sequester intracellular iron) is required for full virulence of *M. robertsii* [184]. Likewise, the ferricrocin of the plant endophyte *Trichoderma virens* is involved in its complicated interactions with plants [185], so the ability of ferricrocin to acquire iron from diverse environments may be another mechanism that facilitates colonization of many different organisms.

In general, the evolution of pathogenicity towards novel hosts or habitats may well be based on traits that were originally developed to ensure survival in the microorganism's original habitat, including soil, plant roots (e.g. IAA) and former hosts (e.g. surviving phagocytosis). Thus, the ability to grow several centimetres down in soil may have preadapted fungi to proliferate in the insect haemolymph with its low oxygen content. The capacity for cross-kingdom host jumps displayed by the *Metarhizium* clade could also depend on them expressing molecules that act upon a wide range of organisms. A study that compared *M. robertsii* with *Aspergillus fumigatus* (a saprotroph with an unusual ability to colonize the respiratory tract) and a plant pathogen (*Haematonectria haematococca*) in their abilities to degrade and use host-derived macromolecules (cockroach cuticle, plant cell walls, horse lung polymers, porcine mucin, hyaluronic acid) found that each fungus secreted a range of enzymes (proteases, phosphatases, phospholipase A2, phospholipase C, phosphodiesterase and esterase), that are common toxic components of bacteria as well as reptile and invertebrate venoms [186]. These widely distributed enzymes all show activity against plant, insect and human tissues, and could provide fungi with the versatility to exploit many environments. It is notable that many common opportunistic human pathogens have a soil-borne phase, including *Aspergillus* species; for instance, *A. fumigatus* is an important pathogen of humans, whereas *Aspergillus flavus* has an even wider host range including plants and insects [187]. These fungi usually grow in decaying vegetation, but many opportunistic bacteria with a capacity for cross-kingdom infection inhabit the rhizosphere, and use similar or even identical functions for beneficial interactions with plants and virulence in humans [188]. Factors that contribute to rhizosphere fitness include the ability to use root exudates as nutrient sources or, more generally, ecological and nutritional versatility [189], a property that opportunist pathogens, particularly broad host range *Metarhizium* strains, have in abundance.

SMs are exemplars of molecules known to have targets in hosts belonging to different kingdoms. A multitude of biosynthetic pathways have been uncovered by *Metarhizium* genome sequences [102,190]. These include pathways likely responsible for known *Metarhizium* chemistries (e.g. destruxins, cytochalasins, ovalicin), and pathways similar to those in other fungi but with candidate products not yet known in *Metarhizium* (e.g. diketopipearzine or resorcylic acid lactones). Based on genomic sequence data indicating that certain *Metarhizium* species have the capacity to produce lysergic acid-derived ergot alkaloids, it was experimentally confirmed that they do, but only during insect colonization and not in plants [191]. Ergot production by the plant-inhabiting Clavicipitaceae is well documented and famously causes serotonergic overstimulation of the central nervous system in animals [192]. *Metarhizium* species are not noted for modifying the behaviour of their hosts, unlike some members of the Ophiocordycipitaceae that co-opt sleep behaviour in insects to cause summit disease e.g. ‘zombie ants’ [193]. Some other pathways in *Metarhizium* genomes are so unique that the molecules they produce cannot yet be predicted [190], highlighting both that genome sequencing is an important tool for bioprospecting, and our fragmentary understanding of how SMs are involved in the interactions of these fungi with other organisms [102]. However, of the known infection-promoting factors, many are toxins that directly target the most conserved cellular components such as the cytoskeleton (e.g. cytochalasins) or cellular membranes (destruxins), and potentially could function against diverse hosts. As well as being well known for their insecticidal activity, destruxins show antimicrobial, antiviral, antiproliferative, cytotoxic and immunosuppressive properties. Likewise, the antibiotic helvolic acid produced by broad host range *Metarhizium* species only [102,194] is a common product of endophytic and plant pathogenic fungi [195].

Some other capabilities appear unique to *Metarhizium* isolates but may preadapt them for various habitats. Filamentous fungi grown in a polluted environment can transform or degrade hazardous compounds, which may be an adaptive survival strategy under severe conditions [196]. Nonylphenol (4-NP) is a xenobiotic classified as an endocrine disrupting compound and poses severe threats to soil ecosystems and human health [197]. *Metarhizium* is reported to have a unique capacity to perform numerous mono- and dihydroxylation reactions both in the aliphatic chain and the 4-NP aromatic ring that completely degrade 4-NP in the environment [198]. The richness of *Metarhizium* as a resource of novel chemistries means that it is often bio-prospected by medical and industrial scientists. A case in point, scientists looking for compounds for the treatment of skin disorders found that *Metarhizium* produces 2-hydroxytyrosol (2-HT), a powerful inhibitor of tyrosinases (phenoloxidases), and previously only known as a synthetic compound. The resulting publication [199] resembles many others on *Metarhizium* metabolism in not mentioning its natural history. It is likely that the capacity to produce 2-HT, and perform some of its other unusual chemistries, evolved in *Metarhizium* to defend against the melee of toxic phenolic compounds and melanizing reactions that comprise an important component of insect antimicrobial defences. *Metarhizium robertsii* certainly has many adaptations to resist this toxicity, including producing a metalloprotease that degrades host phenoloxidases inhibiting melanization activities [200], and secreting its own phenoloxidase to oxidize soluble phenolics to less toxic insoluble melanin [201]. Also, in contrast to proteases from non-pathogens, *Metarhizium* proteases are resistant to melanizing mixtures [202]. It appears that *M. robertsii* attempts to manipulate the melanization response to promote its own growth and survival, although our understanding of these processes is incomplete.

## 9. Econutrition as a controlling factor in *Metarhizium* lifestyle choices

*Metarhizium* strains show a continuum in specialization to insects ranging from: (1) qualitative, characterized by the inability of the pathogen to infect many hosts; (2) quantitative, the pathogen has lower performance on most hosts, and (3) generalist strains able to kill a panel of insects. Multi-host generalist strains of *M. robertsii* and *M. brunneum* have greater nutritional versatility than specialists, colonize plant roots and are facultative entomopathogens, whereas the assumption is that specialized strains are probably dependent upon insect hosts for reproduction. The ability of a generalist strain to attack diverse insects does not rule out adaptations to exploit nutrients on cuticles of frequently met hosts. Homopteran bugs produce secretions rich in sugars that supplement endogenous nutrients on the cuticle. Unlike broad host range lines isolated from Coleoptera that require low levels of complex nitrogenous nutrients to induce appressoria (infection structures), many homopteran-derived lines also produce appressoria in glucose medium [172,203]. Indicative of rapid pathogenic adaptation, even very closely related generalist strains isolated from coleopteran and hemipteran insects show this difference, which is mediated by cyclic AMP [203].

It is noteworthy that formation of infection structures by nematophagous and entomopathogenic fungi depends on both the nutritional status of the fungus and the presence of host chemistries [51,204]. Thus, incorporation into the soil via organic manures of easily accessible nutrients reduces the parasitic activity of *P. chlamydosporia* [205]*.* In the laboratory, it was shown that *M. robertsii* spores applied to an insect are not infectious when provided with a supplementary nutrient source [51], which may in part be because many of the enzymes required for virulence are subject to catabolite repression i.e. only produced when the fungus is nutritionally deprived [206]. Similarly, the *M. robertsii* adhesin MAD2 involved in attachment to the root is upregulated by nutrient deprivation [207], as is the subtilisin Pr1A that is produced in both insect cuticles and plant root exudate [171]. An invertase gene deletion mutant of *M. robertsii* showed reduced catabolite repression and increased root colonization [122].

Evidently, from the perspective of *Metarhizium*, its beneficial associations with plants and virulence to insects are simply means of establishing a nutritional relationship with these hosts. It seems likely that the differential stability of insects and plants as nutrient sources has resulted in *Metarhizium* being much more exploitative of insects. Insects are small transitory nutrient sources, and many of the causes of their high background mortality rate, e.g. predation, will likely terminate the pathogen as well. This will usually select for rapid exploitation of the insect host. Some insect hosts can potentially disperse the fungus long distances, which could have selected for attenuation of virulence and hence improved dispersal in those specialist strains that take a long time to kill. However, sporulation and hence transmission only occur after the fungus has killed the insect. By contrast, plants provide a large potentially long-lived habitat, and no requirement for the host to die in order for the fungus to propagate. Instead, a flourishing plant will provide a bigger and more stable habitat, potentially providing selection for a fungus that can further nourish and protect the plant.

## 10. Sexuality and host range

*Metarhizium* species are studied because of their interesting biology that informs on fungal associations with many other organisms, and because of their long-recognized utility as pest control agents. Many times, basic and applied research dovetail. For example, the frequency of outbreeding is important information affecting diversity and virulence, and will impact the effectiveness of a particular transgenic biocontrol strain, as recombination may allow the spread of transgenes from transgenic *Metarhizium* strains. If the fungus is strictly clonal, its application and tracking will be relatively straightforward. Thus, sex, or the lack thereof, plays an important role in pathogen strategies and the risks associated with genetic engineering. Molecular phylogenetic analyses of *Metarhizium* have shown that sexually reproductive *Metarcordycep* forms are distributed among clades comprised of *Metarhizium* isolates assumed to be reproducing asexually [28,29,107]. The requirements for sexual reproduction in the genus remain enigmatic, but observations suggest that sexual reproduction, as evidenced by teleomorphic forms (note that a teleomorph is the sexual stage of a fungus), is restricted to a narrow range of host insects in geographically restricted regions [107,208]. By contrast, asexual forms tend to occupy extensive geographical distributions and a broader spectrum of niches, particularly the soil environment [172,208]. Other teleomorph fungal genera also have much narrower host ranges compared to their asexual anamorph forms prompting the interesting question of how closely related fungi can differ so fundamentally in ecology depending on sexual state [209].

Sex establishes genetic variation by combining genetic information from two parental lineages. Before genetic information is transferred to the progeny meiotic recombination generates novel combinations of existing alleles. Meiotic recombination is therefore considered an important driver for adaptation. It, therefore, seems counterintuitive that having a wide range of hosts should be associated with asexual reproduction which should limit genetic variation. However, generalists appear to be principally adapted to their ecological habitat, being most abundant in rhizospheric soils, indicating that their root association provides the most frequently encountered resource. The strategies of infection, virulence and suppression of host defences used by generalists tend to target features that are common in many insects, and frequently involves overpowering the host by using numerous enzymes and toxins with adverse effects on many diverse species. By contrast, specialization to a particular insect host usually involves a very abundant above ground feeder (e.g. plant hoppers, caterpillars, grasshoppers, cockroaches etc.) which separates the fungus from its root associations. The insect becomes the commonest resource and therefore principal source of selective pressure. If specialized *Metarhizium* strains usually complete their entire life cycle on a single compatible host, then sexual or parasexual reproduction will be limited to strains with the set of effectors (virulence factors) that allow infection of the same host. Thus, host specialization could establish a barrier preventing gene flow between strains specialized to distinct hosts, leading to species formation as described for plant pathogens [210]. Specialists usually do not produce toxins and take longer to kill than generalists striking a constant balance between virulence and evading/resisting host immunity, thus exhibiting a more sophisticated form of pathogenicity.

A caveat to associating sex with these differences is that not observing sex is not by itself reliable evidence it does not happen, and most work on the evolution of fungal sex has principally focused on its presumed consequences in terms of recombination or the lack of it. Fortunately, long-term asexuality and sex each leave genomic signatures so what has not been observed can now be inferred. Analysis of the genomes of multiple *Metarhizium* species lacking known teleomorphs revealed that they in fact exhibit diverse reproductive modes that strongly influence genome evolution and are linked as cause or effect with pathogenic strategies [105]. *Metarhizium* species resemble most other entomopathogenic members of the three hypocrealean families (Clavicipitaceae, Cordycipitaceae and Ophiocordycipitaceae) in being heterothallic with haploid genomes that carry only one of the mating type (*MAT*) loci. They are thus potentially outcrossing fungi as they require a haploid partner with a compatible *MAT* locus to complete the sexual cycle. Most isolates in the ARSEF collection of the USDA group within the currently defined limits of the closely related *M. pingshaense, M. anisopliae, M. robertsii* and *M. brunneum* (the ‘PARB’ clade). None has yet been confirmed to produce a sexual state, but both the MAT1 and MAT2 idiomorphs have been identified in different isolates of each PARB species, indicating the ongoing potential for sexual reproduction [32]. Signs of sex in supposedly asexual species include footprints of repeat-induced point mutations (RIP), a genome defence mechanism specific to fungi, occurring only during meiosis on repeated sequences. The consequences of RIP are that repeated DNA segments, such as those that would result from the retrotransposition or the duplication of a gene, are inactivated by mutations. Calculations of RIP indices indicated that RIP occurs in narrow host range *M. album* and *M. acridum*, but not in the broad host range PARB species [20]. This suggests retention of meiosis in specialists, even when their sexual stages have not been verified with any observed production of meiotic forms.

If sex occurs in generalist PARB *Metarhizium* species, despite the absence of RIP, it has no notable impact on their population structure, which is largely clonal [9,211,212]. This suggests that even if cryptic mating occurs it may generate progeny fitter than the ubiquitous clonal lineages too rarely to render mating biologically significant. Despite this, a population genetic analysis showed that in addition to clonality, recombination occurred via parasexuality within reproductively isolated groups (i.e. vegetative compatibility groups, VCGs) that function as cryptic species [213]. The parasexual cycle starts with fungi capable of performing anastomosis (the fusion of encountering hyphae) allowing exchange of nuclei to form a heterokaryon, i.e. the advantages of having multiple genomes instead of just one [214]. Sometimes, as these nuclei mix in the cytoplasm they undergo haploid nuclear fusion (karyogamy), resulting in diploid cells. Instead of undergoing meiosis, the vegetative cells continue dividing mitotically, and haploidy is restored by random chromosome loss resulting in cells with unique combinations of chromosomes from either parent nucleus. Many fungi that have never been observed to mate in their natural environment are still capable of parasexuality [215,216]. Furthermore, it has been shown that the thin-walled blastospores *Metarhizium* produces in insect haemolymph may facilitate genetic exchange between isolates co-infecting an insect [217]. Generalists also have a life cycle that entails a soil-dwelling phase where there is considerable potential for different genotypes to mix. However, compared to specialists, generalists such as *M. robertsii* have many more heterokaryon incompatibility protein (HET) domains aborting parasexuality [20]. Thus, asexual *M. robertsii* lineages can only anastomose with practically identical isolates in the same clone (VCG) that are thereby parasexually compatible [172,218]. In nature, green or red fluorescent protein-tagged isolates of *M. robertsii* strain 2575 showed parasexuality producing unstable diploids expressing both fluorescent markers with haploidy being restored by loss of chromosomes through mitosis [9]. That *M. robertsii* strain 2575 undergoes parasexual recombination with itself indicates that recombination can occur between cells of the same mating type. Parasexuality did not occur between *M. robertsii* 2575 and several other strains of *M. robertsii* that were tested in the laboratory [9], consistent with outcrossing by parasexuality being very limited in phylogenetic breadth.

That raises the question as to what purpose does parasexuality serve. A virulent *Metarhizium* produces about 5 × 10^6^ spores per *Drosophila* cadaver [219]. It is estimated that a single gene will be mutated at a frequency of 1 in 6 × 10^4^ fungal conidia [220], so most spores will carry a mutation in a *Metarhizium* genome of about 10 000 genes, and if these mutations are randomly distributed, the conidia from a single cadaver will provide a library of mutations representing much of the genome. Assuming adaptive mutations are rare they will usually be in separate conidia. The ‘Vicar of Bray’ hypothesis (or Fisher–Muller Model) suggests that sex produces individuals of higher fitness by combining adaptive mutations that arise in separate lineages into one genome. Otherwise, the two asexual lineages will compete with each other (so-called clonal interference) [221]. Parasexuality could therefore be an adaptation to combine adaptive mutations in lineages that lack a sexual cycle.

Intriguingly, therefore, generalist genotypes where habitat selection and not host insect selection drive the population structure have lost or almost lost meiosis, but potentially retain parasexuality to prevent clonal interference. Other genotypes with evolutionary histories of insect host specificity have retained meiosis and recombination, as indicated by either active RIP or overt sexual teleomorph (*Metacordyceps*) states. This implies that the plasticity of sexual reproduction is at the forefront of evolution of host range in *Metarhizium* species. Similarly, unlike the teleomorph *C. militaris*, which is specific to lepidopteran pupae, the closely related *B. bassiana* lacks active RIP and has a wide host range [11]. Likewise, the broad host range plant pathogen *Fusarium oxysporum* lacks RIP, unlike cereal specialists such as *F. graminearum* [222], suggesting that this pattern is common in related fungi with diverse host preferences. It is noteworthy that the genomes of *Metarhizium* species reveal chromosomal rearrangements that establish lineage-specific genomic regions, but they also have very extensive regions of synteny in spite of differences in host range, RIP, the number of transposable elements (TEs) and more than 50 Ma of separation [10]. This suggests that clonal reproduction is helping conserve genomic configurations that are well adapted for growth in the host and environment. By contrast, there are no obvious syntenic relationships between the genome structures of *B. bassiana* and *C militaris* [11]. Frequent genetic and/or chromosomal recombination resulting from sexual reproduction has reorganized the genome structure of *C. militaris*.

Clearly, for entomopathogenic fungi, sexual reproduction is beneficial in some situations, but not always, which is why both ways of reproduction still exist. To address why this is the case there is a need to collect data from natural populations and conduct experimental studies to test different hypotheses. However, the Red Queen hypothesis (from Lewis Carroll's *Through the Looking Glass*, in which Alice must run as fast as she can just to stay in place), is usually evoked in these circumstances. It proposes that in evolutionary arms races between pathogens and their hosts, both species must continually adapt in response to each other. Thus, a newly evolved pathogen virulence mechanism will be negated over time by a newly evolved host immune mechanism, and vice versa. According to the Red Queen, sexual reproduction confers species variability and a faster generational response to selection by making offspring genetically unique. Consequently, co-evolutionary interactions between a host insect and its specific pathogen may select for sexual reproduction in both species.

As previously mentioned, broad host range *Metarhizium* and *Beauveria* species are unlikely to have engaged in a strict coevolutionary arms race as they have diverse hosts. That still leaves the question as to why they either lost meiosis or it is very rare (assuming asexual strains evolved from sexual ones). The rice pathogen *Magnaporthe oryzae* reproduces sexually at its centre of origin in Asia but outside that centre reproduces as clones [223]. These clones are highly abundant and may be examples of sampling bias from successful invasive genotypes that will eventually crash and be replaced by the next clone [224]. That could be a model for *Metarhizium*. Nevertheless, *Metarhizium* species have been extensively sampled almost worldwide over many decades, and these investigations have clearly delineated sexual and parasexual populations that differ in lifestyle, with clonal lineages that are old enough to have become very widely dispersed. For example, one clonal population of generalists ranges throughout Brazil into Colombia [172]. There are many potential explanations for the evolutionary shifts from sexual to asexual/parasexual reproduction in generalist *Metarhizium*s, although little empirical data. Fungal sex often involves the production of a dormant spore that is particularly resistant to harsh conditions, and this may not be required by a fungus sheltering in an insect or plant. Meiosis and formation of fruiting structures is also expensive in time and resources compared to mitosis. However, the commonly accepted paradigm is that asexual development allows rapid propagation through large numbers of cheaply produced spores that colonize, persist and spread. Whereas the advantage of sexual development is to generate genetic diversity to accelerate adaptation to novel and changing environments [225] which in the case of *Metarhizium* species includes host insects.

Thus, organisms that reproduce both sexually and asexually tend to switch to sex under stressful conditions, in which case investment in sex should reflect their degree of adaptation to, and thus stress caused by, current environmental conditions. That is exemplified by the saprophytic fungus *Aspergillus nidulans* which undergoes asexual development in favourable conditions but favours sexual development in harsh conditions for which its current gene combination may not be adapted [226]. If relevant to *Metarhizium* species, this implies that the genetic associations built up by past selection will remain favourable for longer in generalist *Metarhizium* forms selected to a plant/soil environment than they will in pathogens specialized to a particular insect(s). This is consistent with a need for rapid changes in gene combinations to keep pace with a coevolving host. However, broad host range *Metarhizium* forms, although apparently asexual, are characterized by high genetic diversity, consistent with their ability to exploit very diverse conditions. Asexual *Metarhizium* species often live in more diverse environments than sexual morphs, and must be adapted to deal with a broad vista of environmental stresses. Furthermore, during infection, pathogenic fungi endure a battery of host-associated stresses including osmotic and oxidative stresses [5], and behavioural changes such as fever [227]. As reviewed by Lovett & St. Leger [228], stress has therefore played an important part in adaptive evolution of asexual *Metarhizium* species to insect, soil and root conditions. While numerous well-known fungal oxidative, nitrosative and heat shock responses are induced when conidia germinate on insect cuticles [10], some of *Metarhizium*'s stress responses are unique among fungi. These include deployment of a small eubacteria-like cold shock protein (CRP1) that protects cells against freezing and increased *M. robertsii*'s virulence to caterpillars, probably by protecting against reactive oxygen species generated as a host defence mechanism [96]. It is likely that the pleiotropic nature of many *Metarhizium* stress-related genes is related to the complexity of stresses *Metarhizium* cells confront in their diverse environments [228].

A related factor to be considered is the effective population sizes of generalist and specialized *Metarhizium* species. Models that account for population sizes have found that sex and recombination evolve much more readily in small populations [229,230]. According to the models, asexuality will be selected for in ‘infinitely large populations' because variation is readily generated by mutation and easily maintained by selection within these populations. Conversely, with few individuals in a population, selection over a few generations will erode variation (particularly in haploids), so sex and recombination will be selected for as allowing genes residing in different unrelated individuals (i.e. not just within biotypes like parasexuality) to be brought together, thereby producing new genotypic combinations upon which selection can act. Clones of generalists are frequently highly abundant and widely dispersed, perhaps approximating the models ‘infinitely large populations’, whereas specialists are often rare and localized.

There may also be circumstances peculiar to generalist entomopathogens that causes sex to be selected against. RIP is incompatible with gene duplication events, so its absence is consistent with expanded gene families and more TEs in the *B. bassiana* and *M. robertsii* genomes, relative to *C. militaris* and *M. acridum*. It is possible, therefore, that sex was selected against because meiosis was selected against as losing meiosis, and therefore RIP, was a prerequisite for generalists expanding gene families. A generalist lifestyle in *Metarhizium* species also coincides with an increase in HET genes and transposons, suggesting that they coevolve with a broad host range, presumably also as a consequence of loss of RIP. HETs restrict gene flow between fungi, and their accumulation in generalist *Metarhizium* species will likely therefore reinforce vegetative incompatibility. Asexual *Aspergillus* species also have more HET genes than those that reproduce sexually which suggests there could be a disadvantage for fungi with a sexual cycle to have such genes [231]. Another reason could be the proposed function for heterokaryon incompatibility in limiting the spread of detrimental cytoplasmic or nuclear elements [232]. Tests showed that heterokaryon incompatibility indeed limits the horizontal transfer of dsRNA mycoviruses to within vegetative compatibility groups of *Metarhizium* [218]. At least in *Aspergillus nidulans*, mycoviruses are excluded from sexual spores so *A. nidulans* has an extra option to get rid of parasitic elements through its sexual cycle reducing the importance of heterokaryon incompatibility [231]. This has not been studied in teleomorphs of *Metarhizium*. Also, not confirmed in *Metarhizium* species, in some fungi heterokaryon formation, and therefore parasexuality, requires genetic identity at all HET genes. In *Neurospora*, the mating-type (MAT) genes are also HET genes and control both sexual compatibility and heterokaryon compatibility, although the former requires that the mating types are different, and the latter requires that the mating types are identical [233]. If also true in *Metarhizium* species, then two isolates could either do parasexuality or sexuality, not both.

Sexual reproduction is frequently described as if it is mysterious in myriad ways. To date, we have little empirical data to base our speculations on, but *Metarhizium* and its sexual relations provide an interesting model for looking at why sex is maintained, and why it might be beneficial. There are still many ambiguities in *Metarhizium* sexuality to be addressed. For the specialist *Metarhizium* species that retain cryptic sex lives the most pressing questions include, how often do these species mate in nature, how are sexual cycles regulated and do they always occur in co-infected hosts? In which case how are sexual cycles regulated by host cues? For generalists generating largely clonal populations, it is generally assumed that genes will decay if the pathway in which they act is no longer functional or adaptive. Yet the PARB species contain MAT loci and most of the suite of genes needed for sexual development, even though they restrict access to this mode of reproduction. We previously reported that unlike *C. militaris* and other fungi with sexual stages, *B. bassiana* and *Metarhizium* species lack Spo11 [11], a conserved recombinase that generates double-stranded DNA breaks required for meiotic recombination (but not RIP) in diverse eukaryotes [234]. Its absence could contribute to an infrequent sexual cycle [11] and indicate progressive mutational decay of a process that is no longer protected by selection. For this review we ran the *Neurospora crassa* Spo11 sequence through a BLAST database containing all the *Metarhizium* genomes and identified a domain of the type found in IIB DNA topoisomerase and Spo11. Most of these were listed as hypothetical proteins but experimental verification is needed to confirm the status of Spo11 activity in these fungi.

The scarab specialist *M. majus* is diploid and heterozygous at multiple loci [172], suggesting that a double infection with compatible isolates produced a mating attempt that failed to complete meiosis. This raises the question of whether these fungi are ever able to complete the sexual cycle [208]. However, some *Candida* species manage to have a sexual cycle although lacking several mating genes and genes for meiosis [235]. Because *C. albicans* mating involves diploid partners, their fusion generates a tetraploid cell. Instead of meiosis, the tetraploid cell undergo a parasexual process of concerted chromosome loss to generate diploid and aneuploid progeny; genetic recombination is observed in a subset of parasexual progeny and is dependent on Spo11 [236]. This indicates that there are parallels between the parasexual cycle and a conventional meiosis, and that in either process genetic recombination involving crossing over requires Spo11. Production of recombinant progeny in the basidiomycete *Cryptococcus neoformans* is also dependent on Spo11 [237], so the status of Spo11 in *Metarhizium* species may provide an explanation for their tight genomic synteny, as there is clearly strong selection for retention of genomic configurations.

The genes responsible for parasexuality are not known, so it is possible that many elements of the parasexual cycle in PARB *Metarhizium* species require sex cycle genes, explaining their conservation. It is also likely that many sex cycle genes are pleiotropic. Thus, although listed as a sex cycle gene, the gene for EsdC is involved in cell wall morphology and is expressed by *M. robertsii* during growth on root exudates [171]. Even the MAT genes, MAT1-1 and MAT1-2, besides regulating pheromone and pheromone receptor genes, affect other genes not involved directly in the mating process [238]. Conversely, the lack of fertility in fungi with no known sexual stage can be due to changes in one or more of numerous genes that cause female sterility (inability to produce fruiting structures) [238]. This suggests that a sexual cycle can be easily lost in a fungus reproducing asexually over many generations.

## 11. Mechanisms for rapid adaptation in asexual *Metarhizium* species

There is an expectation that asexual populations will adapt more slowly than sexual ones [239], but ‘sex or no sex, evolutionary adaptation occurs regardless' [240]. Several mechanisms, including mutations and horizontal gene transfer (HGT, i.e. the movement of genetic material between distant organisms) have been proposed for the diversity seen in asexual *Metarhizium* populations. These are mostly haploids, and haploids adapt faster than diploids in asexual populations because of much greater impact of single mutations [241]. It is also possible that the chemical stressors in insects or the root exudate are themselves mutagenizing, although this has not been tested. However, a clonal lineage of *M. robertsii* strain 2575 showed adaptive changes to a new habitat within a few years of transfer, despite recurrent genetic bottlenecks and lack of recombination with locally well-adapted strains [9]. This was due, at least in part, to regulatory mutations effecting expression of cell wall and stress response genes, consistent with evolutionary theory that rapid evolution often occurs by ‘old genes-new regulation’. There was no evidence for transposition contributing to variation during the five-year experiment, but it potentially could do so in an historical context. The outlier *O. sinensis* is again informative. In contrast to most insect pathogens *O. sinensis* contains two compatible MAT loci in the genome and is sexually self-fertile, i.e. homothallic [16]. Hu *et al.* [16] proposed that inbreeding is an adaptation by *O. sinensis* to its small population size resulting from a very specialized lifestyle and the extreme environmental conditions in its small geographical range. The RIP mechanism is dysfunctional in *O. sinensis*, and as noted previously, *O. sinensis* has a hugely inflated genome mediated by the accumulation of repetitive elements, which has contributed to the large number of retrotransposed and fragmented pseudogenes in the genome. The massive proliferation of TEs may provide a trade-off between advantages of increased genetic variation independent of sexual recombination and deletion of genes dispensable for its specialized pathogenic lifestyle [16]. As *O. sinensis* has lost many genes for expanding its host range, future transitions away from its current lifestyle seem unlikely, indicating that while retrotransposition may facilitate rapid adaptation, it may also contribute to stable host interactions.

HGT can also accelerate adaptation to a new environment, and multiple independent HGT events in *Metarhizium* species, occurring over many millions of years, have played an important role in genome evolution and host-range expansion [242]. Many of the older HGT gene products are proteases or lipid carriers involved in cuticle penetration and exploiting cuticular proteins and epicuticular lipids for nutrition. Some of these genes were acquired by ascomycete fungi ancestral to the *Metarhizium* clade, and retained by some or all of the clade, whereas orthologues were subsequently lost from multiple related lineages. This presumably reflects lack of selection for their retention in fungi that do not breach insect cuticles. An interpretation consistent with a phylogenomic survey of fungal gene family evolution which suggested that individual protease genes have been lost many times independently in different lineages, and that flux of genes is an ongoing process [200]. The older HGT genes are all most similar to sequences in soil bacteria consistent with the ancestor of *Metarhizium* having an ecological niche in soil. The more recently acquired genes include sequences in soil- and insect-dwelling bacteria continuing an ecological niche overlap [242]. Not surprisingly, therefore, insect hosts are also a source of adaptive traits, including the striking example of a sterol transporter that affected pathogen evolution by allowing *Metarhizium* to compete for sterols with their insect hosts [194]. Recent acquisitions at the origin of the late-evolving PARB clade include gene clusters encoding SMs that were laterally acquired from other fungal genera [20]. For example, the destruxin gene cluster may have been acquired 15 Ma by *Metarhizium* lineages that were broadening their host range [20]. At least five of the most recently acquired HGT genes from bacterial and arthropod sources may have contributed to host-range expansion in PARB species, as their heterologous expression in the acridid specialist *M. acridum* expanded its host range [242]. Although the mechanism of HGT in *Metarhizium* is unknown, transposons have been implicated in other fungi, especially if they colonize a common host [243], and the PARB clade is enriched in transposons. Leal-Bertioli *et al.* [217] report that following double infection of chrysomelid beetles with two isolates of *M. anisopliae*, only one or the other isolate was detected in 52 of 53 dead hosts. Both genotypes were detected in the 53rd host insect, and one single-spore isolate from that had a novel band by RAPD analysis. Transfer of repetitive DNA (e.g. a transposon), rather than the parasexual cycle is suggested since this would explain new bands rather than just segregation of parental bands [217].

## 12. Concluding remarks

A wealth of molecular techniques has been used to document the diversity and prevalence of *Metarhizum* species, in many of their guises, within soil, plant, nematode and insect populations. The *Metarhizium/P. chlamydosporia* clade, likely evolved from plant root associates and generalists, retain the ability to colonize the plant hosts of soil-dwelling herbivorous insects and nematodes, the proximity providing a plausible scenario for the evolutionary switch from root associate to a dual lifestyle. It is likely that *Metarhizium* isolates have additional associations with soil organisms, including amoeba and other fungi, providing further benefit to their plant habitats. Flexible shifting between multiple hosts may be able to make use of pre-existing traits (receptors, signal transduction pathways, enzymes) rather than new pathogen adaptations. However, adaptation may be required for sustained transmission between new hosts, as *Metarhizium* strains can kill some insects without producing spores on cadavers. The opportunism and evolvability of the genus, and their worldwide distribution, makes *Metarhizium* one of the commonest eukaryotes in most terrestrial environments, probably reaching their greatest abundance in pasture soils [173].

*Metarhizium* species that are principally soil-dwelling root associates appear to be adapting to their ecological habitat rather than available insect hosts. Their overall strategy is to be highly exploitative of short lived transitory hosts, but beneficial to plants that provide a stable environment. Their ability to kill a wide range of insects maximizes protection to the plants and provides a frequently encountered resource of nitrogen that the fungus trades with the plant for carbon. Recent intensive and focused sampling has identified many new *Metarhizium* species that are pathogens to a narrow host range of insects, and unlike generalists frequently retain meiosis and a sexual cycle [199]. For the most part, specialist *Metarhizium* species target insects that live above ground, separating the fungus from its ancestral root habitat, and compared to generalists, they seem to be rare and live in geographically restricted regions. However, as many of these regions are in understudied parts of the world, sampling bias could be a factor if sexual *Metarhizium* species are not evenly distributed.

Comparative genomics has shown that gene families associated with pathogenesis have expanded in *Metarhizium* species compared to saprophytes and plant pathogens, and the generalists overall have more of these enzymes than narrow host range species. Specialists have a larger number of rapidly evolving genes compared to generalists, consistent with rapid evolution of existing protein sequences during an evolutionary arms race with their hosts. The strategies used by generalists typically involve overpowering the host by using numerous enzymes and toxins that have adverse effects on many insects. To achieve this, generalists have undergone extensive gene duplications, and are particularly enriched in proteases and SMs [105]. These gene duplications were permissible because of the loss of RIP, a DNA defence mechanism that occurs during meiosis. It is possible therefore that the loss of a sexual stage by generalists is not altogether related to advantages or disadvantages of producing genetically diverse progeny, but because of selection against meiosis, and in particular its concurrent process RIP.

However, many theories have been produced to account for the predominance of sexuality in eukaryotes, and many of these theories can be applied to the retention of parasexuality in generalists and the sexual cycle in specialists, and either linked as cause or effect with their pathogenic strategies. Overall, the key distinction between the sexual cycle and parasexuality in *Metarhizium* species seems to be that the former allows unrelated individuals of different mating types to exchange genetic material, whereas parasexuality limits the exchange to the same biotype (and hence mating type). Parasexuality fulfils one of the advantages of a sexual cycle in providing a mechanism for generalist *Metarhizium* within a biotype to combine beneficial mutations. This should speed spread of beneficial mutations because they will not risk being excluded by interfering mutations in other lineages of the same clone. However, compared to a sexual cycle it decreases the risk of recombining an already well-adapted combination of genes.

Overall then, *Metarhizium* speciation provides a model for the evolution of endophytes, host preference, specificity and virulence. Many new molecules and functions have been discovered that underpin *Metarhizium* disease or are involved in other ecologically relevant traits, and with comparative genomics have improved understanding of the nature, timing and architecture of genomic changes governing local adaptation. Recent studies have addressed outstanding evolutionary questions that are particularly important for biocontrol agents and address fundamental, yet poorly understood issues in molecular evolution by asking about the roles different kinds of mutations play in adaptation, whether adaption to new environments occurs because of changes to a few genes or many, and the timescale at which evolutionary processes happen. The high level of synteny between *Metarhizium* genomes, irrespective of their lifestyle, suggests that point mutations may be as important as genomic rearrangements. Rapid adaptation by a generalist strain to a new soil and plant environment frequently involved beneficial mutations in regulatory elements, and these mutations accumulated quickly in an asexual population, even with recurrent bottlenecks [9]. Many phenotypic differences between humans and chimpanzees are also accounted for by regulatory mutations against a very similar genetic background [244], consistent with this being a ubiquitous phenomenon underlying rapid evolution irrespective of whether reproduction is asexual, parasexual or sexual.

## Data accessibility

This article has no additional data.
